# Improved phylogeny of brown algae *Cystoseira* (Fucales) from the Atlantic-Mediterranean region based on mitochondrial sequences

**DOI:** 10.1371/journal.pone.0210143

**Published:** 2019-01-30

**Authors:** Carolina Bruno de Sousa, Cymon J. Cox, Luís Brito, Maria Madalena Pavão, Hugo Pereira, Ana Ferreira, Catarina Ginja, Lenea Campino, Ricardo Bermejo, Manuela Parente, João Varela

**Affiliations:** 1 Centro de Ciências do Mar, Universidade do Algarve, Faro, Portugal; 2 Universidade dos Açores, Faculdade de Ciências e Tecnologia, Ponta Delgada, Açores, Portugal; 3 CIBIO-InBIO, Centro de Investigação em Biodiversidade e Recursos Genéticos, Universidade do Porto, Campus Agrário de Vairão, Vairão, Portugal; 4 Global Health and Tropical Medicine, Instituto de Higiene e Medicina Tropical, Universidade Nova de Lisboa, Lisboa, Portugal; 5 Departamento de Biología- Área de Ecología, Facultad de Ciencias del Mar y Ambientales, Universidad de Cádiz, Puerto Real, Cádiz, Spain; 6 Irish Seaweed Research Group & Earth and Ocean Sciences Department, Ryan Institute and School of Natural Sciences, National University of Ireland, Galway, Ireland; 7 CIBIO-Açores, Centro de Investigação em Biodiversidade e Recursos Genéticos, InBIO Laboratório Associado, Pólo dos Açores, Departamento de Biologia, Universidade dos Açores, Ponta Delgada, Portugal; Fred Hutchinson Cancer Research Center, UNITED STATES

## Abstract

*Cystoseira* is a common brown algal genus widely distributed throughout the Atlantic and Mediterranean regions whose taxonomical assignment of specimens is often hampered by intra- and interspecific morphological variability. In this study, three mitochondrial regions, namely cytochrome oxidase subunit 1 (COI), 23S rDNA (23S), and 23S-tRNAVal intergenic spacer (mt-spacer) were used to analyse the phylogenetic relationships of 22 *Cystoseira* taxa (*n* = 93 samples). A total of 135 sequences (48 from COI, 43 from 23S and 44 from mt-spacer) were newly generated and analysed together with *Cystoseira* sequences (9 COI, 31 23S and 35 mt-spacer) from other authors. Phylogenetic analysis of these three markers identified 3 well-resolved clades and also corroborated the polyphyletic nature of the genus. The resolution of *Cystoseira* taxa within the three clades improves significantly when the inclusion of specimens of related genera was minimized. COI and mt-spacer markers resolved the phylogeny of some of the *Cystoseira* taxa, such as the *C*. *baccata*, *C*. *foeniculacea* and *C*. *usneoides*. Furthermore, trends between phylogeny, embryonic development and available chemotaxonomic classifications were identified, showing that phylogenetic, chemical and morphological data should be taken into account to study the evolutionary relationships among the algae currently classified as *Cystoseira*. The resolution of *Cystoseira* macroalgae into three well supported clades achieved here is relevant for a more accurate isolation and identification of natural compounds and the implementation of conservation measures for target species.

## Introduction

*Cystoseira* (Fucales, Heterokonta) brown algae are key elements of the marine seascape along warm-temperate North African and European coasts [1–4]. They form marine forests with a complex three-dimensional structure and provide habitat for other algae, invertebrates and fish [5–8], playing a key role in the determination of biodiversity patterns and ecosystem functioning [6]. Currently, many *Cystoseira* taxa are undergoing a strong demographic decline attributed to both local and global pressures [9–11]. Moreover, it has been suggested that this loss of biodiversity might be caused by the sensitivity of these macroalgae to increased water turbidity, eutrophication and pollution [12–14], as a consequence of the increasing anthropogenic activity near the Atlantic and Mediterranean coastal areas [10, 11]. Changes in the distributions and abundance of various species are also expected as a consequence of climate change [15, 16]. Because of the ecological importance of assemblages dominated by *Cystoseira* and the deterioration of their populations during the past decades, the Mediterranean species of this genus are protected under the Barcelona Convention (Annex II, COM/2009/0585 FIN) and reforestation has been proposed as a management action to improve the conservation status of these macroalgae [14, 17].

The importance of the genus *Cystoseira* is further underscored by the observation that its members produce several potentially bioactive metabolites such as terpenoids, fatty acids, triacylglycerols, steroids, phlorotannins, and polysaccharides [18, 19]. Indeed, antioxidant, anti-inflammatory, antiproliferative, antifungal, antiviral, antibacterial and antiprotozoal activities have been reported to occur in *Cystoseira* algae with increasing frequency [20–26]. This wide range of bioactivities detected in extracts of these algae might be explained by the bio- and chemical diversity of the genus [27, 28].

The accuracy of the taxonomic identification of the biomass used for the isolation and identification of natural compounds is, however, an important issue concerning the reproducibility and reliability of the results as well as for the implementation of conservation measures for the target macroalgae [29]. Taxonomic classification within the genus *Cystoseira* is challenging and controversial [30, 31]. Erroneous taxonomical assignments are frequent due to the wide morphological variability of *Cystoseira* individuals, in addition to there being many species that are still undergoing active speciation and hybridization [32–34]. This has become especially apparent due to frequent conflicts between classification of specimens based on morphology and molecular data. Chemotaxonomic classifications based on the presence or absence of specific chemicals (e.g. meroterpenoids) have also been attempted [18, 28, 35–37]. In addition, analysis of the global chemical profile and the lipophilic composition of five *Cystoseira* taxa from Brittany have been found to be in agreement with the phylogenetic relationships established by the ITS2 region [37]. However, congruence between morphology, chemistry and molecular taxonomy at the species level is yet to be achieved [34], and the results obtained so far have not fully resolved the *Cystoseira* phylogeny [37].

Several authors have previously attempted the elucidation of the relationships within this genus and with related genera using phylogenetic methods [34, 38–40]. Analysis of Fucales (Phaeophyceae) Kylin based on large subunit (LSU) and small subunit (SSU) of the ribosomal DNA sequences led to the merging of the Cystoseiraceae De Toni and Sargassaceae Kützing families [41]. The mitochondrial 23S ribosomal subunit (23S) proved to be useful for defining genera in the Fucales [34] and in addition a set of 10 additional mitochondrial, plastid and nuclear markers has also been used to investigate the evolutionary history of brown algae at the ordinal level [42]. Other analysis including also organellar markers revealed that the genus *Cystoseira* was composed of at least six distinct, but clearly polyphyletic, evolutionary lineages. However, only 3 lineages (see below) were eventually classified as separated genera [34]. Based on morphologic, embryonic development characters and genetic data, several members of the genus were reclassified as belonging to the genera *Sirophysalis* (Tropical Indo-West-Pacific), *Polycladia* (eastern Indian Ocean) and *Stephanocystis* (North Pacific) [34]. All other *Cystoseira* taxa, despite forming at least three separate Northeastern Atlantic-endemic clades, retained the original classification. Currently, the genus *Cystoseira* encompasses approximately 40 taxa, the majority of which occurs in the Mediterranean and Atlantic-Mediterranean regions [43, 44]. However, to date, full infrageneric resolution of the genus and their position among related Sargassaceae genera has not been established. Therefore, the taxonomy of the *Cystoseira* species is still unclear.

The mitochondrial gene coding for cytochrome oxidase subunit 1 (COI) is a well-known molecular tool used for the identification of different metazoan taxa [45–47]. Although the COI gene was used in the study of red [48] and brown algae [49–51], the utility of this marker for the infrageneric identification of *Cystoseira* individuals has not been evaluated so far. With the purpose of improving the resolution of the *Cystoseira* species identification and clarify their phylogenetic relationships, a comprehensive study combining sequence information on the cytochrome oxidase subunit 1 (COI), 23S rDNA (23S), and 23S-tRNAVal intergenic spacer (mt-spacer) was undertaken. The results of this study confirm the polyphyly of the genus, which was resolved into 3 well supported clades by using sequence information on the protein-coding COI gene.

## Material and methods

### Ethics statement

We state that no specific permissions were required for the taxa sampled in this work. The samples were taken from public sea places and not from any national park or protected area. The Portuguese Foundation for Science and Technology approved this type of research by supporting our research projects CCMAR/Multi/04326/2013 and PEst-E/EQB/LA0023/2011.

### Sampling

Overall, this study includes 93 samples of *Cystoseira* and 210 sequences belonging to 31 species of the Sargassaceae family (*Cystoseira*: 22 taxa; *Bifurcaria*: 1 species; *Polycladia*: 2 species; *Sirophysalis*: 1 species; *Stephanocystis*: 4 species and *Turbinaria*: 1 species). A detailed list of samples and sequence information is provided in the supporting information (S1 Table). However, it should be noted that, in this study, “Cystoseira” is a term of convenience, which includes all taxa previously classified as belonging to this genus and which have not been redefined by Draisma et al. [31].

**Fig 1:**
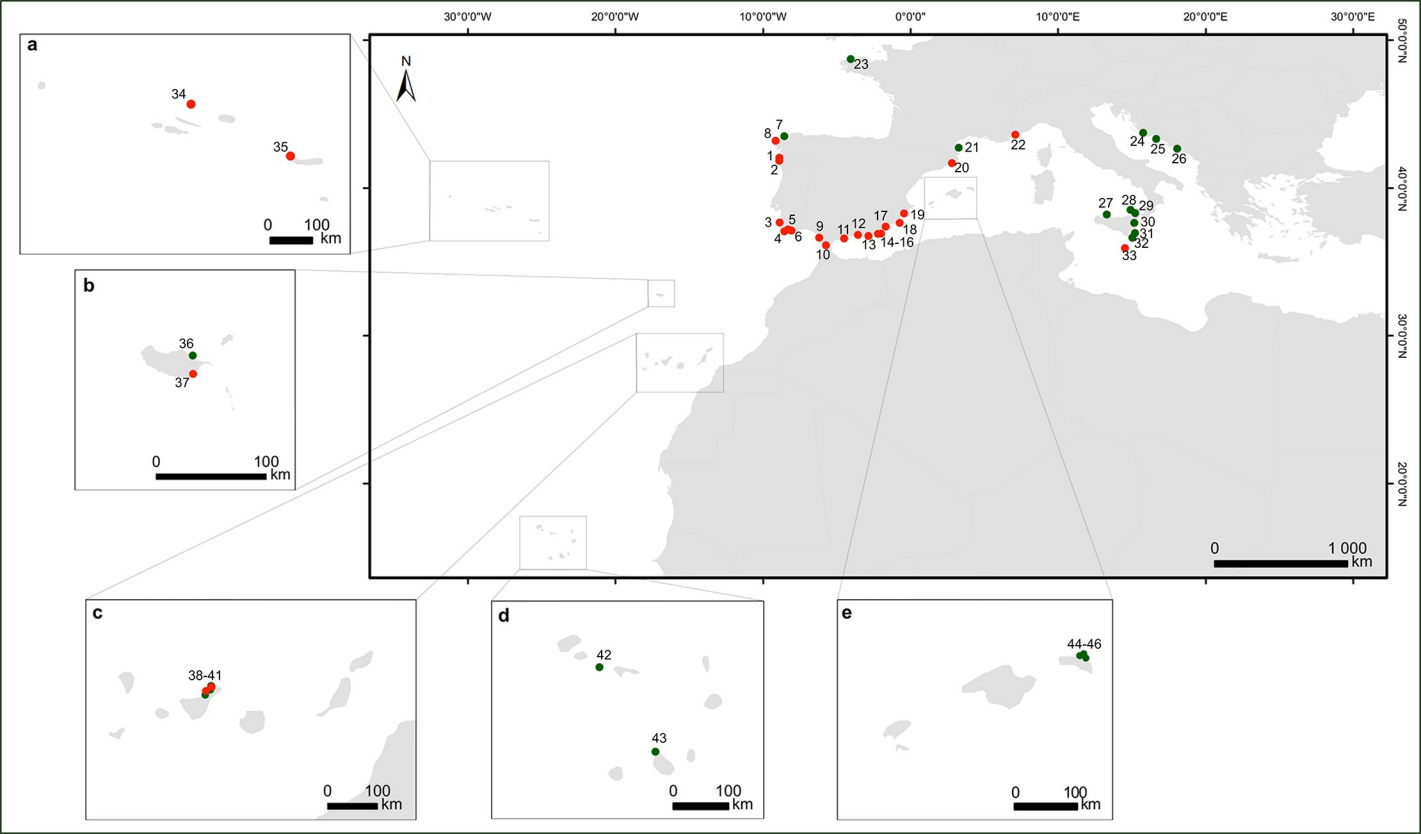
Geographical distribution of the *Cystoseira* samples used in this study. Green dots represent GenBank sequences and the red dots data obtained in this study. The boxes show the archipelagos of ^a^Madeira, ^b^Azores, ^c^Canary, ^d^Cape Verde and ^e^Balearics. Locations and sampling number marked as red dots are: ^1^Moledo (*n* = 1); ^2^Areosa (*n* = 2); ^3^Odeceixe (*n* = 1); ^4^Manuel Lourenço (*n* = 4); ^5^Olhos de Água (*n* = 9); ^6^Arrifes (*n* = 1); ^7^A Coruña (*n* = 4); ^8^Santa Mariña (*n* = 2); ^9^Santibañez (*n* = 2); ^10^El Mirlo (*n* = 3); ^11^Calaburras (*n* = 1); ^12^Herradura (*n* = 1); ^13^Guardias Viejas (*n* = 1); ^14^La isleta del Moro (*n* = 2); ^15^El Playazo (*n* = 2); ^16^Las Negras (*n* = 1); ^17^La Serena (*n* = 1); ^18^Cabo de Palos (n = 1); ^19^Santa Pola (n = 1); ^20^Blanes (n = 3); ^21^Cote Vermeille (*n* = 2); ^22^Pointe I´lette (*n* = 1); ^23^Santec (*n* = 2); ^24^Prvic Island (*n* = 1); ^25^Brac Island (*n* = 1); ^26^Dubrovnik city area *n* = 1); ^27^Capo Gallo (*n* = 1); ^28^Aeolian Island (*n* = 2); ^29^Capo Milazzo (*n* = 1); ^30^S. Maria la Scala (*n* = 3); ^31^Marzameni (*n* = 4); ^32^Capo Passero (*n* = 2); ^33^Xghajra (*n* = 1); ^34^Carapacho (*n* = 1); ^35^Ponta dos Mosteiros (*n* = 2); ^36^Porto da Cruz (*n* = 1); ^37^Caniço (*n* = 2); ^38^Bajamar (*n* = 1); ^39^Mesa del Mar (*n* = 4); ^40^Punta del Hidalgo (*n* = 6); ^41^Tacoronte (*n* = 1); ^42^Branco island (*n* = 1); ^43^Tarrafal Bay (*n* = 1); ^44^Cala Viola de Llevant (n = 1); ^45^La Llosa d'en Patro Pere (*n* = 1); ^46^Illots de Tirant (*n* = 1); ^47^Cala Mica (*n* = 2); ^48^Illa d'es Porros (*n* = 1). For further information about the location of sample points, please refer to the S1 Table.

Fifty-nine samples of *Cystoseira* sp. (*n* = 55) and *Bifurcaria bifurcata (n* = 4) were collected along the Atlantic and Mediterranean coasts (Fig 1), and mtDNA markers were specifically amplified. The samples, collected by the authors or kindly provided by expert colleagues, were morphologically classified using the taxonomic characteristics following Gómez-Garreta et al. [38] and Cormaci et al. [52]. Guiry and Guiry [44] was used as an additional reference for taxonomic validity. After washing with tap water, biomass was silica-dried and stored at room temperature for DNA extraction. Vouchers of the studied specimens were deposited in the herbarium of the University of Algarve (https://www.ualg.pt/pt/content/algu)—Index herbariorum code: ALGU. Additional vouchers were deposited in the herbarium of the Marine Biotechnology Group of the Centre of the Marine Sciences (MarBiotech / CCMAR).

Additional *Cystoseira* sequences (9 COI, 31 23S and 35 mt-spacer) and other Sargassaceae species (3 COI, 8 23S and 8 mt-spacer) publicly available in the GenBank database at the National Center for Biotechnology Information (NCBI) were included in the analysis [53] (Fig 1). Additionally, sequences from 4 species of the Fucaceae family (4 COI, 4 23S and 4 mt-spacer) were also obtained from GenBank and used as outgroup.

### DNA extraction, amplification and sequencing

Genomic DNA was extracted from 5–10 mg of the silica gel-dried algal tissue using the method described by Doyle and Doyle [54]. The primers for amplification of the COI and 23S fragments were described by Lane et al. [55] and Draisma et al. [34], respectively. Primer pairs for amplification of the mt-spacer fragment were designed specifically for this study. Primer information, such as locus names, nucleotide sequences, and references are provided in Table 1.

**Table 1:** Molecular markers used in this study. Locus name and target region, forward and reverse primer sequences, and references.

Target region	Primer	Sequence	References
**23S**	mt23S-FB	5'-AGCGTAACAGCTCACTGACCTA-3'	[31]
	mt23S-RB	5'-CTGTGGCGGTTTAAGGTACGGTT-3'	
**mt23S(partial)-IGS-tRNALys-IGS-tRNAVal**	tRNALys-FW	5'-GGGGTGAAAAATATCACTTTGA-3'	This study
	tRNALys-RV	5'-AACCCAAGACCCTCGGATTA-3'	
**COI**	GazF2	5'-CCAACCAYAAAGATATWGGTAC-3'	[51]
	GazR2	5'-GGATGACCAAARAACCAAAA-3'	

Mitochondrial 23S and mt-spacer were PCR-amplified in a final volume of 20.5 μL reactions containing 5 μL of genomic DNA (~10 ng/mL), 4 μL 5×PCR Buffer, 4 μL dNTP mix (1 mM of each dNTP), 2 μL 25 mM MgCl_2_, 0.6 μL Taq DNA polymerase (GoTaq DNA Polymerase, Promega), 0.5 μL of 10 μM 23S forward (mt23S-FB) and reverse (mt23S-RB) primers or 0.25 μL of 10μM mt-spacer forward (mt-spacer-F) and reverse (mt-spacer-R) primers. COI amplifications were perfomed in a 12-μL mix containing 2 μL of genomic DNA, 1.25 μL 5×PCR Buffer, 0.6 μL dNTP mix (1 mM of each dNTP), 1.25 μL 25 mM MgCl2, 0.1 μL Taq DNA polymerase, 0.25 μL of 10 μM COI forward (GazF2) and reverse (GazR2) primers. Amplifications were performed using an Applied Biosystems 2720 Thermal Cycler with the following conditions: 95°C for 6 min; 10 cycles of 95°C for 30 s, 64°C (decreasing 0.5°C per cycle) for 30 s, 72°C for 60 s; 35 cycles of 95°C for 30 s, 59°C for 30 s, 72°C for 60 s; and a final elongation step of 10 min at 72°C for the 23S and mt-spacer fragments; for COI, samples were incubated at 95°C for 2 minutes; 5 cycles of 95°C for 30 s, 45°C for 30 s and 72°C for 1 min; 35 cycles of 95°C for 30 s, 46.5°C for 30s and 72°C for 1 min; and a 72°C elongation step for 7 min. PCR amplicons were screened for specific fragment size on 2% agarose gel electrophoresis and subsequently purified using a EZNA MicroElute Cycle-Pure Kit (Omega Bio-Tek, USA) purification kit. Amplified fragments were sequenced using the Sanger method at the Molecular Biology Core Laboratory, Centre of Marine Sciences (Algarve University, Faro), in an 3130XL Genetic Analyzer (Applied Biosystems) using PCR primers in cycle sequencing reactions.

### Sequence validation and genetic diversity

New sequences generated from amplicons obtained from both strands were compared with GenBank data using BLASTn [56] to determine whether the biological source was a Sargassaceae alga. GenBank accession numbers of the sequences are indicated in S1 Table. Sequences were also organized in two datasets: one including only sequences from individuals of the *Cystoseira* genus, and the other comprising the same data plus those from the Sargassaceae and Fucales families.

The 23S and mt-spacer sequences were aligned with the CLC Sequence Viewer V.7.6.1 (Quiagen), using the default settings. For COI, sequences were aligned with transAlign software [57] using ClustalW multiple sequence alignment [58]. Alignments were further inspected with CLC Sequence Viewer V.7.6.1 and manually improved before a final curation step with Gblocks v.0.91b software [59] available at the Phylogeny.fr web service [60]. Gap positions within the final blocks option were allowed and a maximum of 8 contiguous non-conserved positions were considered with a minimum block length of 5 nucleotides (nt). The concatenated matrix was obtained using Seaview v.4.5.3 [61]. The number of polymorphic and phylogenetically informative sites of the aligned sequences were estimated for each marker using DnaSP v.5.10.1 software [62]. Haplotype identification was carried out for each mitochondrial marker using this software.

### Evolutionary divergence and phylogenetic relationships

Genetic distance analysis was used to investigate inter- and intraspecific evolutionary divergence between *Cystoseira* sequences. Pairwise-sequence distances were estimated using the Kimura 2-parameter model [63] with MEGA5 software [64]. The rate variation among sites was modelled with a gamma distribution (shape parameter = 6). All ambiguous positions were removed for each sequence pair. Phylogenetic analysis was carried out using Maximum likelihood (ML) and Bayesian inference (BI). The substitution models that best fit the data were selected using MrModeltest2 v.2.3 [65] and PAUP* v.4.0b10 [66] by applying the Akaike information criterion (AIC) [67]. The substitution models selected were: GTR+I+Γ4 [general time-reversible (GTR) model with a proportion of invariant sites (I) and among-site rate variation modelled by a discrete gamma distribution with 4 categories (Γ4)] for 23S, HKY+I+G [Hasegawa-Kishino-Yano model (HKY)] for COI and GTR+Γ4 for the mt-spacer.

ML analysis was performed using RAxML v.7.0.4 [68] with 400 bootstrap replicates, assuming the best-fitting models. Posterior probabilities were determined by Markov Chain Monte Carlo (MCMC) sampling in MrBayes v.3.1.2 [69, 70]. MrBayes analyses were also conducted using the best-fitting models, using 6 chains for 10,000,000 generations, sampling every 1,000th generation, and default settings for the remaining options. Convergence of the MCMC and burn-in were determined through the analysis of the generations vs. log probability plot using the trace analysis tool TRACER v1.6 (http://beast.bio.ed.ac.uk/Tracer). The initial burn-in step discarded 20% of the samples.

After inferring the phylogeny, the topological congruence between gene trees was visually assessed for each marker (COI, 23S, mt-spacer). Subsequently, the sequences obtained for the three markers were concatenated and analysed by ML and BI as described before. ML and BI best consensus trees for each marker dataset (COI, 23S, mt-spacer, and concatenated COI-23S-mt-spacer) were generated and edited with the graphical viewer FigTree v.1.3.1 [71].

The genetic relationships between haplotypes were also investigated by means of a Median-Joining (MJ) network constructed with the NETWORK version 4.5.10 software [72].

## Results

### Alignment characterization

Overall, sequences from 93 *Cystoseira* samples belonging to 22 taxa from the Atlantic (Macaronesian and Iberian Peninsula south and west coasts) and the Mediterranean (Adriatic, Alboran, Balearic and Tyrrhenian seas) regions were included in this study (Fig 1). Among these, the 55 *Cystoseira* samples collected generated 135 new sequences representing a sequencing success of 87.3% (48 sequences), 78.2% (43 sequences) and 80.0% (44 sequences) for COI, 23S and mt-spacer loci, respectively.

**Fig 2:**
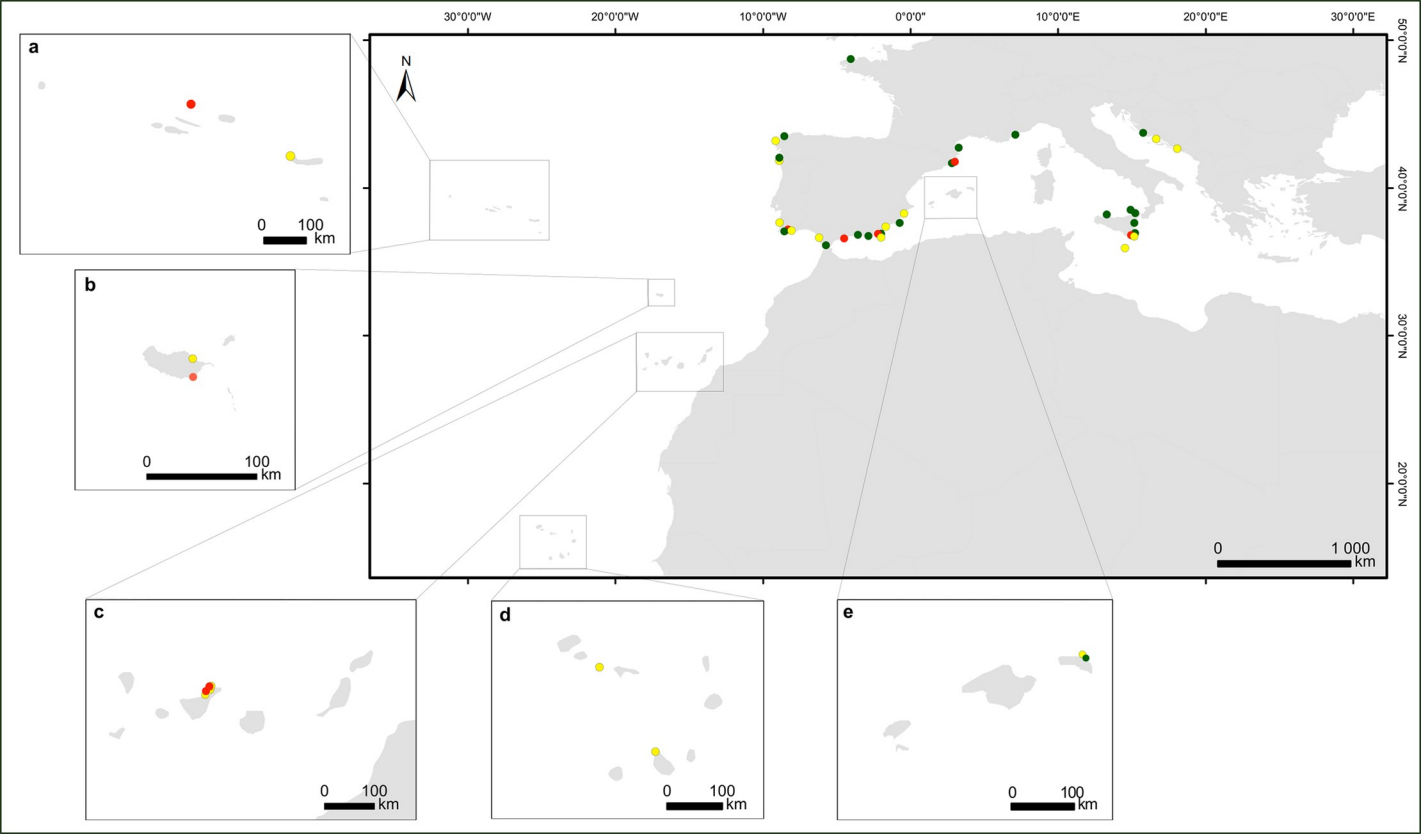
*Cystoseira* groups defined by the phylogenetic analysis. Green dots represent the taxa belonging to the Group I (*Cystoseira tamariscifolia*, *C*. *amentacea* and *C*. *amentacea* var. *stricta*, *C*. *funkii*, *C*. *mediterranea*, *C*. *brachycarpa* var. *brachycarpa*, *C*. *brachycarpa*, C. *barbatula*, *C*. *zosteroides*, *Cystoseira* RB105 and *Cystoseira* sp. 1); yellow dots represent the taxa belonging to the Group II (*C*. *mauritanica*, *C*. *barbata* f. *aurantia*, *C*. *montagnei* and *C*. *montagnei* var. *tenuior*, *C*. *barbata*, *C*. *nodicaulis*, *C*. *granulata*, *C*. *elegans*, *C*. *squarrosa*, *C*. *usneoides*, *C*. *baccata*, *C*. *abies-marina*, C. sonderi, *Cystoseira* sp. 2 and *Cystoseira* sp. MP14); red dots represent the taxa belonging to the Group III (*C*. *compressa* and *C*. *compressa* subsp. *pustulata*, *C*. *humilis*, *C*. *humilis* var. *myriophylloides* and *C*. *foeniculacea*, *Cystoseira* sp. MP1, *Cystoseira* sp. MP2 and *Cystoseira* sp. MP31).

**Fig 3:**
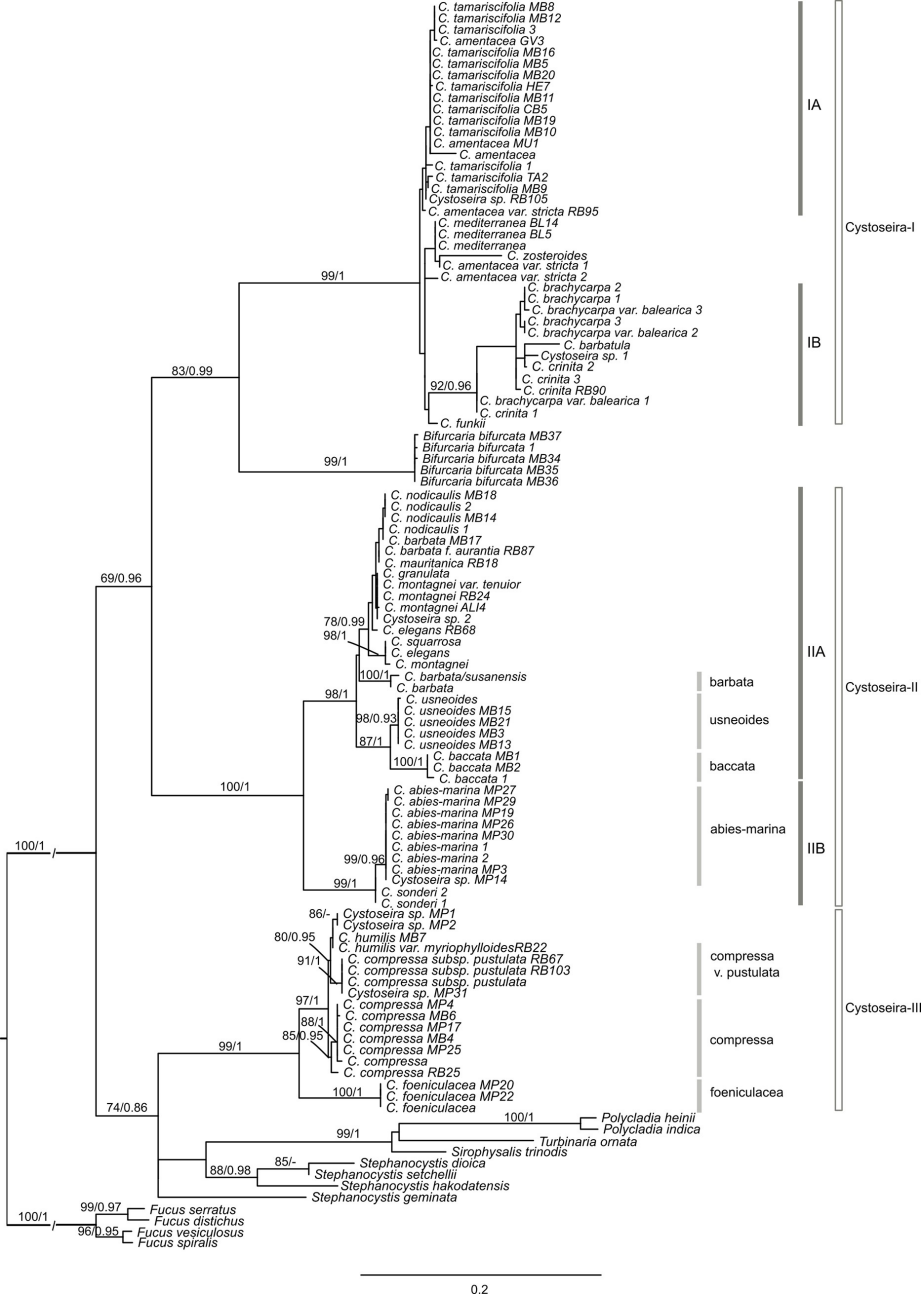
Maximum likelihood phylogenetic tree obtained with RAxML and based on the concatenated COI-23S-IGS sequences of samples from the Sargassaceae family. Values on the branches represent maximum likelihood bootstrap support values (≥ 75) on the left, and Bayesian posterior probabilities (≥ 90%) on the right.

**Fig 4:**
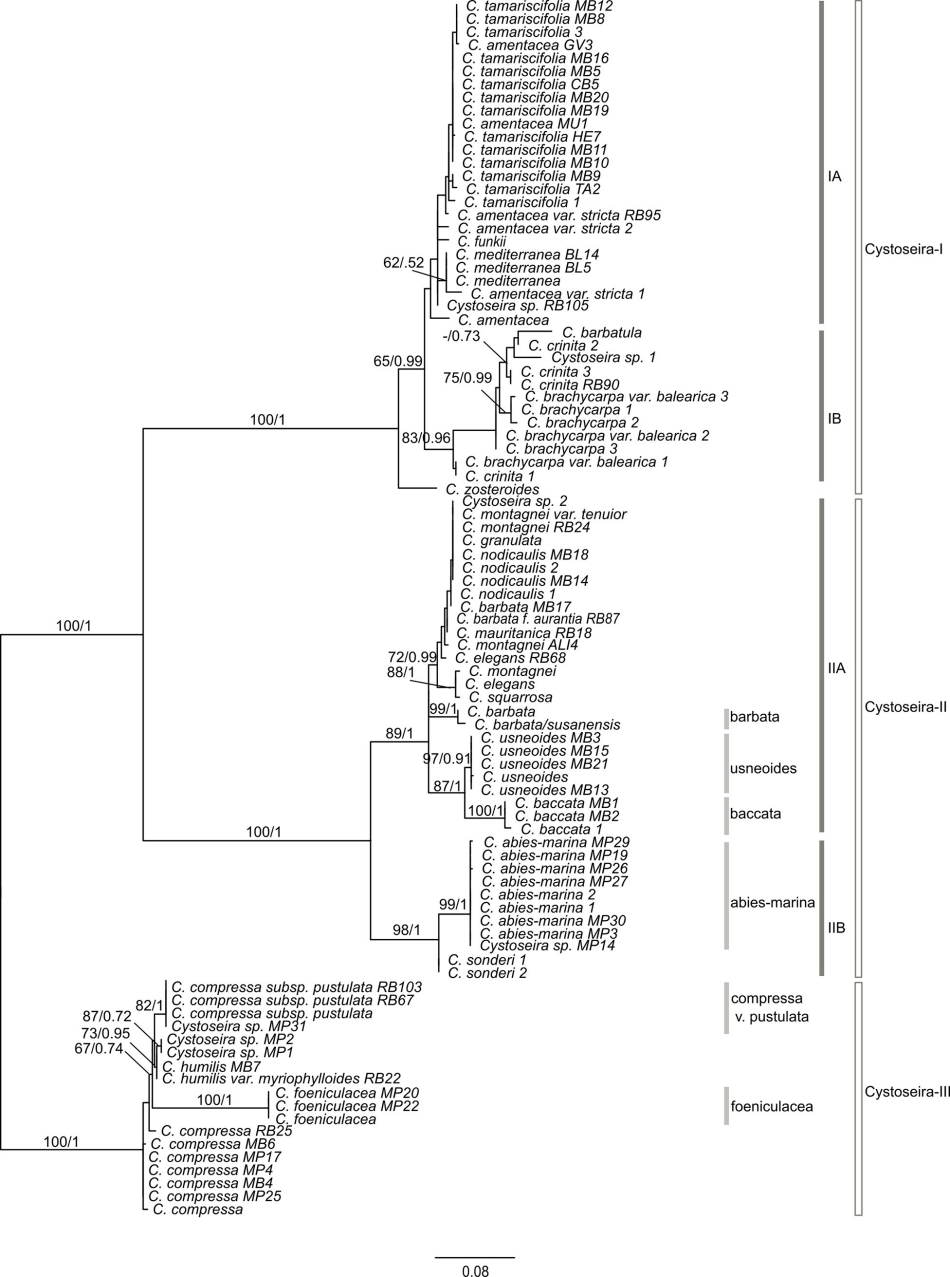
Maximum likelihood phylogenetic tree obtained with RAxML and based on the concatenated COI-23S-mt-spacer sequences of samples from the *Cystoseira* genus. Values on the branches represent maximum likelihood bootstrap support values (≥ 75) on the left, and Bayesian posterior probabilities (≥ 90%) on the right.

The conjoint analysis of *Cystoseira* sequences obtained in this study and from GenBank (57 COI, 74 23S and 79 mt-spacer sequences) resulted in alignments with 656, 391, 258 nt for COI, 23S and mt-spacer, respectively. Upon phylogenetic analysis, three lineages (Cystoseira-I, -II, -III) with support values close to the maximum (BS = 100; PP = 1) were identified (Figs 2–4 and S1–S8 Figs). Detailed information of the alignment results obtained for each marker and phylogenetic group is shown in Table 2. Longer alignment lengths and higher number of conserved positions were observed for COI (656 nt; 86.1%) and 23S (391 nt; 81.7%) loci, and the lowest for the mt-spacer (258 nt; 52.7%).

**Table 2:** Number of *Cystoseira* taxa and samples included in this study. Alignment characteristics (with gaps) are also shown for each marker and phylogenetic group.

	Parameters	All taxa	Group I^1^	Group II^2^	Group III^3^
**COI**					
	**Taxa**	13	3	7	3
	**Number of samples (sequences)**	58	18	25	15
	**Alignment length (nt)**	656	656	656	656
	**Conserved sites**^a^	565 (86.1%)	648 (98.7%)	604 (92.0%)	535 (81.6%)
	**Polymorphic sites**	114 (17.4%)	8 (1.2%)	52 (7.9%)	30 (4.6%)
	**Singleton variable sites**	4 (0.6%)	1 (0.2%)	4 (0.6%)	2 (0.3%)
	**Parsimony informative sites**	110 (16.7%)	7 (1.1%)	48 (7.3%)	28 (4.3%)
**23S**					
	**Taxa**	20	8	9	3
	**Number of samples (sequences)**	73	31	29	13
	**Alignment length (nt)**	391	391	391	391
	**Conserved sites**^a^	317 (81.7%)	335 (85.7%)	331 (84.7%)	352 (90.0%)
	**Polymorphic sites**	61 (15.6%)	25 (6.4%)	22 (5.6%)	10 (2.6%)
	**Singleton variable sites**	5 (1.3%)	10 (2.6%)	0 (0%)	1 (0.3%)
	**Parsimony informative sites**	56 (14.3%)	15 (3.8%)	22 (5.6%)	9 (2.3%)
**mt-spacer**					
	**Taxa**	21	7	11	3
	**Number of samples (sequences)**	79	35	33	11
	**Alignment length (nt)**	258	258	258	258
	**Conserved sites**^a^	136 (52.7%)	183 (70.9%)	141 (54.6%)	168 (65.1%)
	**Polymorphic sites**	62 (24.0%)	25 (9.7%)	34 (13.2%)	18 (7.0%)
	**Singleton variable sites**	4 (1.6%)	7 (2.7%)	5 (1.9%)	2 (0.8%)
	**Parsimony informative sites**	58 (22.5%)	18 (7.0%)	29 (11.2%)	16 (6.2%)

^1^Group I—*Cystoseira tamariscifolia*, *C*. *amentacea*, *C*. *amentacea* var. *stricta*, *C*. *funkii*, *C*. *mediterranea*, *C*. *brachycarpa*, *C*. *brachycarpa* var. *balearica*, *C*. *barbatula*, *C*. *zosteroides* and *Cystoseira* sp. 1

^2^Group II—*C*. *mauritanica*, *C*. *barbata* f. *aurantias*, *C*. *montagnei* and *C*. *montagnei* var. *tenuior*, *C*. *barbata*, *C*. *nodicaulis*, *C*.*granulata*, *C*. *elegans*, *C*. *squarrosa*, *C*. *usneoides*, *C*. *baccata*, *C*. *abies marina*, *C*. *sonderi*, *Cystoseira* sp. 2 and *Cystoseira* sp. MP14

^3^Group III—*C*. *compressa* and *C*. *compressa* subsp. *pustulata*, *C*. *humilis*, *C*. *humilis* var. *myriophylloides* and *C*. *foeniculacea*, *Cystoseira* sp. MP1, *Cystoseira* sp. MP2 and *Cystoseira* sp. MP31.

^a^Percentage calculated relative to the alignment length.

Concatenation of the three loci (COI-23S-mt-spacer) consisted of a 1305-nt alignment with 78% of conserved positions. Depending upon the marker considered, 15.6–24.0% of polymorphic sites (PS) and 14.3–22.5% of parsimony informative (PI) sites were identified (Table 2). Group Cystoseira-II showed the highest number of variable PS (7.9–13.2%) and PI (5.6–11.2%) for all loci, except for the 23S marker, where 6.4% of PS were found (Table 3). Group Cystoseira-III showed the lowest PS (2.6–7.0%) and PI (2.3–6.2%) values for 23S and mt-spacer loci, respectively.

**Table 3:** Comparison of the different *Cystoseira* phylogenetic groups defined in this study with the groups identified by other authors based on genetic, chemical and morphological traits.

Reference	This study	Draisma et al. [34]	Amico [18]^2^	Valls et al. [28]^3^	Piatelli [35]^4^	Amico et al. [27]^5^	Colombo et al. [80]^6^
Type of data	Phylogeny		Chemistry			Morphology	
Taxa^1^	COI, 23S, mt-spacer	23S	Lipophylic, diterpenoid and meroditerpenoid content			Anatomic traits	Embryo germination
***C*. *amentacea***	Cystoseira-IA	Cystoseira-5	VI	IIIB / IIIC	VII	I	I
***C*.* funkii***	Cystoseira-IA	Cystoseira-5	-	-	-	-	-
***C*. *mediterranea***	Cystoseira-IA	Cystoseira-5	VII	IIIB / IIIC	VII	I	I
***C*. *tamariscifolia***	Cystoseira-IA	Cystoseira-5	VII	IIIB / IIIC	VII	I	I
***C*.* barbatula***	Cystoseira-IB	Cystoseira-5	III	IIIA	III	-	-
***C*. *brachycarpa***	Cystoseira-IB	Cystoseira-5	II	II	II	II	I
***C*. *crinita***	Cystoseira-IB	Cystoseira-5	III	IIIA	III	II	I
***C*. *zosteroides***	Cystoseira-IC	Cystoseira-5	IV	IIIB	IV	III	I
***C*. *baccata***	Cystoseira-IIA	Cystoseira-6	V	IIIB	-	VI	II
***C*. *barbata***	Cystoseira-IIA	Cystoseira-6	I	I	III	II	I
***C*. *elegans***	Cystoseira-IIA	Cystoseira-6	V	IIIA / IIIB	V	III	I
***C*. *granulata***	Cystoseira-IIA	-	-	-	-	-	-
***C*. *mauritanica***	Cystoseira-IIA	-	-	-	-	III	-
***C*. *nodicaulis***	Cystoseira-IIA	-	-	-	-	III	I
***C*. *montagnei***	Cystoseira-IIA	Cystoseira-6	V	IIIB	V	III	I
***C*. *squarrosa***	Cystoseira-IIA	-	IV	-	IV	III	-
***C*. *usneoides***	Cystoseira-IIA	Cystoseira-6	IV	-	-	III	-
***C*. *abies-marina***	Cystoseira-IIB	Cystoseira-6	-	-	-	II	-
***C*.* sonderi***	Cystoseira-IIB	-	-	-	-	-	-
***C*. *compressa***	Cystoseira-IIIA	Cystoseira-4	I	I	I	IV-V	III
***C*. *humilis***	Cystoseira-IIIA	Cystoseira-4	I	I	I	IV-V	III
***C*. *foeniculacea***	Cystoseira-IIIB	Cystoseira-4	-	IIIA	III	IV-V	III

^1^Conspecifity of taxa used by different authors [44]: *C*. *amentacea* = *C*. *stricta*; *C*. *brachycarpa* = *C*. *balearica* = *C*. *caespitosa*; *C*. *barbata* = *C*. *susanensis*; *C*. *nodicaulis* = *C*. *granulata*; *C*. *montagnei* = *C*. *spinosa* = *C*. *jabukae*; *C*. *squarrosa* = *C*. *spinosa* var. *squarrosa*; *C*. *foeniculacea* = *C*. *Ergovicii*

^2^Chemical groups based on the meroditerpenoids composition: Group I = no lipophilic secondary metabolites; Group II = linear diterpenoids; Group III = linear meroditerpenoids; Group IV = tetrahydrofurans, furans and pyran ring; Group V = cyclic meroditerpenoids; Group VI = Bicyclo[3.2.0]heptane ring system; Group VII = Rearranged meroditerpenoids

^3^Valls et al.’s chemical groups: Group I—No diterpenoids; Group II—Linear diterpenoids; Group III–Meroditerpenoids: III.A—Linear meroditerpenoids; III.B—Cyclic rneroditerpenoids; III.C—Rearranged meroditerpenoids

^4^Piatelli’s chemical groups on the chemical composition: Group I—no lipophilic secondary metabolites; Group II—linear diterpenoids; Group III—open-chain meroditerpenoids; Group IV—tetrahydrofurans and furans; Group V—cyclopentane ring; Group VI–bicyclo[4.3.0]nonane ring system; Group VII–bicyclo[3.2.0]heptane ring system

^5^Morphological groups based on the receptacle, conceptacle and axis characteristics: Group I = *C*. *ericaefolia* (*C*. *amentacea*, *C*. *mediterranea*, *C*. *tamariscifolia*); Group II = *C*. *crinito-selaginoides* (*C*. *abies-marina*, *C*. *barbata*, *C*. *brachycarpa*, *C*. *crinita*); Group III = *C*. *spinifero-opuntioides* (*C*. *elegans*, *C*. *mauritanica*, *C*. *nodicaulis*, *C*. *montagnei* = *C*. *spinosa*, *C*. *squarrosa*, *C*. *zosteroides*); Group IV-V = *C*. *discors-abratanifolioides* (, *C*. *compressa*, *C*. *foeniculacea*, *C*. *humilis*); Group VI (*C*. *baccata*)

^6^Colombo et al. identified morphological groups based on the embryo characteristics: Group I–Spherical embryo germination and 4 primary rhizoids; Group II—Spherical embryo germination and 4 primary rhizoids and different segmentation sequence; Group III–Ovoid embryo germination with 8 primary rhizoids.

### Evolutionary divergence and haplotype analysis

Interspecific evolutionary divergence of *Cystoseira*, considering only the taxa that have information for all the three markers, ranged from 0.0 to 6.8% in COI, 0.0 to 4.6% in 23S and 0.0 to 14% in the mt-spacer (Table 4 and S2–S4 Tables). The highest level of interspecific variation was observed in the Cystoseira-II (0–14%) group, whereas Cystoseira-I taxa showed the lowest range of genetic distances (0–1.1%) for all markers. Overall, intraspecific variation was lower than the variation observed between species. Intraspecific divergence ranged from 0 to 5.6% in COI, 0.0–2.2% in 23S and 0–3.9% in the mt-spacer. When considering all the samples included in the phylogenetic analysis, the intraspecific divergence increased slightly higher (up to 7.6%), as a result of the greater heterogeneity of the species included. In general, mean genetic distances were greater for mt-spacer, followed by COI and 23S loci.

**Table 4:** Evolutionary divergence between COI, 23S and mt-spacer *Cystoseira* sequences.

Markers—Group	All *Cystoseira* samples		*Cystoseira* with information of the 3 markers*	
	Interspecific	Intraspecific	Interspecific	Intraspecific
**COI**				
** Cystoseira-I**	0.0–1.1	0.0–0.3	0.0–1.1	0.0–0.3
** Cystoseira-II**	0.0–6.8	0.0–5.6	0.0–6.8	0.0–5.6
** Cystoseira-III**	0.0–4.4	0.0–0.6	0.6–4.4	0.0–0.6
**23S**				
** Cystoseira-I**	0.0–4.9	0.0–2.2	0.0–2.3	0.0–2.2
** Cystoseira-II**	0.0–4.6	0.0–1.6	0.0–4.6	0.0–1.6
** Cystoseira-III**	0.3–2.1	0.0–0.3	0.3–2.1	0.0–0.3
**mt-spacer**				
** Cystoseira-I**	0.0–9.6	0.0–7.6	0.0–4.4	0.0–2.6
** Cystoseira-II**	0.0– - 14	0.0–3.9	0.0–14	0.0–3.9
** Cystoseira-III**	0.0–11.4	0.0–1.1	0.4–11.4	0.0–1.1

* samples without species identification were excluded

A total of 16 COI, 26 23S and 37 mt-spacer haplotypes were identified, in 58, 73 and 79 *Cystoseira* sp. individuals, respectively. The greatest haplotype diversity was observed for Cystoseira-I (the total number of haplotypes was 4, 13 and 15 for COI, 23S and mt-spacer, respectively) and–II (7, 7 and 16 haplotypes), whereas Cystoseira-III had the lowest diversity (5, 6, and 6 haplotypes). Several haplotypes were exclusive of each *Cystoseira* group, and the Median-Joining analysis revealed highly congruent networks across markers for each Cystoseira-I, -II and–III groups (S9–S11 Figs). A total of 21 haplotypes out of the 79 found were shared between at least two taxa of the same group. The Cystoseira-I taxa were those with the highest number of shared haplotypes (*n* = 11 for all markers), followed by the Cystoseira-II (*n* = 6) and Cystoseira-III (*n* = 5) taxa. For each *Cystoseira* group, these haplotypes were only shared within sub-groups, which were clearly separated in the networks.

### Phylogenetic analysis

Maximum likelihood and Bayesian inference analyses of the Sargassaceae (Fig 3) and Cystoseira-only (Fig 4) concatenated datasets confirm the subdivision of *Cystoseira* in 3 well-suported clades (Cystoseira-I-III; Figs 3 and 4 and S1–S6 Figs). This subdivision was congruent among analyses of each mitochondrial marker (S3–S8 Figs).

Overall, the Cystoseira-III group, which includes *C*. *compressa*, *C*. *foeniculacea*, *C*. *humilis*, clearly branched off Cystoseira-I (C. amentacea, *C*. *barbatula*, *C*. *brachycarpa*, *C*. *crinita*, *C*. *funkii*, *C*. *mediterranea*, *C*. *tamariscifolia*, *C*. *zosteroides*) and Cystoseira-II (*C*. *abies-marina*, *C*. *baccata*, *C*. *barbata*, *C*. *elegans*, *C*. *mauritanica*, *C*. *nodicaulis*, *C*. *sonderi*, *C*. *montagnei*, *C*. *squarrosa*, *C*. *usneoides*; Table 3). However, these results suggest that Cystoseira-I and -II are more closely related as compared to Cystoseira-III, sharing a common branch with maximum support (BS = 100; PP = 1; Fig 4). Nonetheless Cystoseira-I and -II are paraphyletic when Bifurcaria is included in the analysis, as was observed with the Cystoseira-III taxa that clustered together with other genera from the Indo-Pacific region previously classified as Cystoseira (BS = 74; PP = 0.86), such as *Polycladia*, *Sirophysalis* and *Stephanocystis* [34].

Cystoseira-I could be divided into two subgroups Cystoseira-IA and -IB (Figs 3 and 4). Cystoseira-IA (*C*. *amentacea*, *C*. *funkii*, *C*. *mediterranea*, *C*. *tamariscifolia*) formed a well-supported cluster (BS = 96; PP = 1) using mt-spacer sequences (S7 and S8 Figs), although without significant statistical support in the 23S analysis (S5 and S6 Figs). Within this group, *C*. *mediterranea* formed a cluster that was ML-supported in the COI tree (BS = 99; PP = 0.93; S3 and S4 Figs), while *C*. *tamariscifolia* and *C*. *amentacea* remained unresolved. Subgroup Cystoseira-IB (*C*. *barbatula*, *C*. *brachycarpa*, *C*. *crinita*) was significantly supported in the concatenated datasets analysis (BS = 92; PP = 0.96; Figs 3 and 4); and in the 23S tree, support was highly significant (BS = 99; PP = 1; S5 and S6 Figs). This result suggests that *C*. *brachycarpa*, *C*. *barbatula* and *C*. *crinita* are indeed closely related. In addition, Cystoseira-I taxa clustered together with a well-supported *Bifurcaria bifurcata* cluster (BS = 94; PP = 1; Fig 3 and S1 Fig), confirming that they are sister taxa.

Cystoseira-II branched into two well-supported subgroups, Cystoseira-IIA (BS = 100; PP = 1) and Cystoseira-IIB (BS = 98/99; PP = 1; Figs 3 and 4). This high support is mainly due to the inclusion of the COI and mt-spacer markers (S3 and S4 Figs). Analysis of the concatenated dataset showed that Cystoseira-IIA (*C*. *baccata*, *C*. *barbata*, *C*. *elegans*, *C*. *mauritanica*, *C*. *nodicaulis*, *C*. *montagnei*, *C*. *squarrosa*, *C*. *usneoides*) encompassed two well-resolved taxa, namely *C*. *usneoides* (BS = 98/97; PP = 0.93/0.91) and *C*. *baccata* (BS = 100; PP = 1) (Figs 3 and 4). Maximum support of the *C*. *baccata* clade was also obtained in the COI tree (S3 and S4 Figs), whereas in the 23S tree the branch support values were lower (BS = 89; PP = 0.92; S5 and S6 Figs). *C*. *usneoides* cluster was supported by the ML analysis using the COI (BS = 96; PP = 0.54; S3 and S4 Figs) and 23S (BS = 94; PP = 0.92; S5 and S6 Figs) loci. In addition, Cystoseira-IIA included an unresolved heterogeneous set of taxa (Figs 3 and 4), although the COI locus allowed for the resolution of a *C*. *nodicaulis* cluster (BS = 86; PP = 0.99; S3 and S4 Figs). However, the presence of a well-supported heterogeneous cluster (BS = 98/88, PP = 1) encompassing three sequences acquired from the GenBank and classified as *C*. *montagnei*, *C*. *elegans*, *C*. *squarrosa* from the Adriatic and nearby Sicily Mediterranean coasts was not in agreement with the results of sequences of the same species obtained in the Spanish south Mediterranean coast (S1 Table). Sister to Cystoseira-IIA, Cystoseira-IIB contained *C*. *abies-marina* and *C*. *sonderi* and formed a well-supported cluster (BS = 99/98; PP = 1; Figs 3 and 4), though this topology was not detected in the 23S analysis (S5 and S6 Figs).

Within the Cystoseira-III group, *C*. *foeniculacea* formed a clade with maximum support (BS = 100; PP = 1), sister to *C*. *compressa* and *C*. *humilis* as defined by all markers (Figs 3 and 4 and S1 and S2 Figs). Although without significant support values (BS = 80/67, PP = 0.95/0.74 in Figs 3 and 4, respectively), *C*. *compressa* branched off *C*. *compressa* subsp. *pustulata* and *C*. *humilis*. These results are in agreement with some authors [52,73] that consider *C*. *compressa* subsp. *pustulata* a synonym of *C*. *humilis* var. *humilis*. Therefore, we suggest that the former should be renamed as the latter. These relationships are better defined in the COI trees (S3 and S4 Figs) that suggest the occurrence of three independent clades: *C*. *compressa* (BS = 90, PP = 0.9), *C*. *humilis* (BS = 94, PP = 0.95) and *C*. *compressa* subsp. *pustulata* renamed as *C*. *humilis* var. humilis (BS = 96, PP = 1). The importance of COI to clarify the infrageneric phylogeny and improve the identification of *Cystoseira* samples is well illustrated by the analysis of *Cystoseira* sp. MP2 and *Cystoseira* sp. MP31 individuals. Even though these samples were classified as belonging to the genus *Cytoseira*, morphology alone did not allow the identification of the specimens down to the species level. However, the COI trees obtained in this study strongly suggest that *Cystoseira* sp. MP2 and *Cystoseira* sp. MP31 should be classified as *C*. *humilis* and *C*. *humilis* var. *humilis*, respectively (Figs 3 and 4 and S3 and S4 Figs).

## Discussion

The present study represents a comprehensive survey of the diversity of the genus *Cystoseira*, based on 92 samples from 22 different *Cystoseira* taxa and other Cystoseiraceae. To the best of our knowledge, this is the first study using a combination of COI, 23S and mt-spacer sequences to investigate the phylogeny of the *Cystoseira* genus. This analysis contributed 48 COI, 43 23S and 44 mt-spacer sequences from a wide geographic area (Figs 1 and 2), enlarging significantly the number of sequences available in GenBank. Additionally, emphasis was given to *C*. *tamariscifolia*, *C*. *amentacea* and *C*. *mediterranea*, whose phylogenetic relationships are still poorly clarified.

Compared to previous studies [34,42], the *Cystoseira* sequences obtained here had a relatively low number of phylogenetically informative sites (16.7% PI sites for COI, 14.3% for 23S and 22.5% for mt-spacer). This might be explained by our focus on *Cystoseira* and the limited use of sequences of related genera in order to minimize the number of gaps in alignments of highly variable regions, such as the mt-spacer. Analyses of the interspecific divergence yielded genetic distance values similar to those described for other algae [74–75]. Fucales seem to have low zygote dispersal [76, 77] and, as a result, it is predicted that macrophytes belonging to this order show low intra-population genetic diversity, but larger differentiation among different regional populations [78, 79]. The inclusion of a wider array of closely related genera suggests, however, that Cystoseira-I and Cystoseira-III macroalgae are phylogenetically closer to specimens of other genera (namely *Bifurcaria*, *Polycladia*, *Stephanocystis* and *Sirophysalis*) than to those of Cystoseira-II (Fig 3), making this genus polyphyletic as noted by Draisma et al. [34]. Therefore, our results suggest that from an evolutionary point of view the Atlantic-Mediterranean *Cystoseira*, as currently defined, correspond to distinct groups that should be classified as three different genera.

The comparison of our results with those of other studies, including genetic, chemical and morphological information [18, 27, 28, 34, 35 cited by 36 and 80 cited by 27] (Table 3), led to identification of similarities between taxa of these groups. Phylogenetic results corroborate the polyphyletic nature of the genus *Cystoseira* described previously [34, 40, 41]. A direct correspondence between our classification and that proposed by Draisma et al. [34] was found, namely Cystoseira-I, Cystoseira-II and Cystoseira-III are analogous to Cystoseira-5, Cystoseira-6, and Cystoseira-4, respectively. Moreover, the results of our haplotype analysis were highly consistent with the phylogenetic trees concerning the identification of subgroups within Cystoseira-I, -II and -III (S9–S11 Figs), regardless of their geographical location. For example, the mt-spacer clearly distinguished the Cystoseira IA from -IB, -IIA from -IIB and -IIIA from -IIIB median-joining networks. The same conclusion was reached with the 23S marker, reinforcing the existence of identifiable sub-groups of taxa within the aforementioned groups. Concerning the morphology of some reproductive and support structures (receptacle, conceptacle and axis), the Cystoseira-I cluster matches Groups I and II described by Amico et al. [37]. Moreover, Amico et al. [37]’s Group III, known as “*C*. *spinifero-opuntioides”*, corresponds to the Cystoseira-II taxa of the present work. The only exception was *C*. *zosteroides*, which branches off early in trees either obtained in this study (Fig 4 and S5–S8 Figs) or in those described by Draisma et al. [34], though often without statistical support. Groups IV-V and VI as defined by Amico et al. [37] correspond to Cystoseira-III (*C*. *compressa*, *C*. *humilis* and *C*. *foeniculacea*) algae and Cystoseira-II (*C*. *baccata*), respectively. Group III of Colombo et al. [80], based on criteria related to embryo germination [37], matches the Cystoseira-III taxa (Table 3). However, among the Cystoseira-II taxa different morpho-anatomical traits and types of embryo germination can be found. For example, *C*. *baccata* and *C*. *barbata* were classified as belonging to Amico et al.’s groups VI and II, respectively. Concerning embryo germination, these two taxa were classified in different groups as well, once again confirming the difficulty in finding common traits to define taxa within this group of macroalgae. However, a trend for Cystoseira-II taxa to belong to Amico et al.’s group III and the Colombo et al.’s group I could be observed. For a more detailed description of the morphological traits of these groups, please refer to S5 Table.

Although Draisma et al. [34] discarded any connection between phylogeny and the published chemotaxonomic classifications, a careful comparison between all traits allowed to detect some trends, as also noted by Susini [40]. For example, linear diterpenoids and rearranged meroterpenoids [18, 28 and 35 cited by 36] are exclusive to Cystoseira-I taxa, which have been identified as the most “chemically evolved” group according to the structural complexity of their secondary metabolites [35 cited by 28], in agreement with the results obtained in this phylogenetic study. Unlike Cystoseira-I and–II algae, all Cystoseira-III taxa lack diterpenoids and lipophilic secondary metabolites, being thus defined not by the presence of a given class of chemicals, but by its absence. Similar trends can also be observed at the sub-group level (Table 3). For example, specimens of the Cystoseira-IA and -IB subgroups identified in the present study match chemical Groups VI/VII and Groups II/III, respectively, as described by Amico [18]. Another example would be the fact that Cystoseira-IIB algae are restricted to Amico’s chemical Groups I, IV and V. Interestingly, only *C*. *zosteroides*, which branches early off the remaining Cystoseira-I taxa, shares a similar chemical profile to Cystoseira-IIA algae, namely *C*. *squarrosa* and *C*. *usneoides*. Taken together, these results suggest that there might be a closer relationship between phylogenetic, chemical and morphological classifications than previously thought.

The mt-spacer locus described as having high resolving power for *Fucus* spp. [81] was considered to be useful only at a generic level for Sargassaceae [34] and insufficiently informative to differentiate between the closely related *C*. *montagnei* and *C*. *squarrosa* taxa [39]. Despite these arguments and the high variability of mt-spacer, which can generate large gaps if the choice of taxa to include in the alignment is too divergent, *C*. *barbata*, *C*. *baccata* and *C*. *abies-marina* (Cystoseira-II), and *C*. *foeniculacea* (Cystoseira-III) were resolved from their closest relatives with significant support in mt-spacer trees, which was also supported by the network analysis (S9–S11 Figs).

Even though the authors tried to minimize the inclusion of GenBank sequences assigned to misidentified taxa by including only sequences that were previously used by other authors, incongruence of taxonomic assignment between samples obtained in this study and elsewhere were detected. This applies to the identification of *C*. *montagnei* and *C*. *elegans* collected in the Adriatic Sea and the Alboran Sea, which do not cluster together. Thus, additional sampling targeting these species should be envisaged in future studies, so that this inconsistency is resolved. In fact, the use of more reliable methods for taxonomic assignment, such as the cojoint use of morphological and genetic data to test congruence, should become the norm.

Another question addressed by the present work is the difficulty to distinguish between closely related taxa, namely *C*. *tamariscifolia*, *C*. *amentacea* and *C*. *mediterranea*, based on morphological criteria alone. Morphological plasticity, crypticism and seasonal variability in the appearance of these macroalgae often hinders and, in some cases, even prevents the accurate, unambiguous taxonomical assignment of the samples [30, 31, 82]. Thus, this reinforces the need for novel tools able to differentiate these taxa, especially in places where they coexist [82]. Although the analyses using the three markers under study did not support the resolution of *C*. *tamariscifolia* from *C*. *amentacea*, the COI trees show a well-supported cluster of *C*. *mediterranea*. Moderately high interspecific divergences with low intraspecific variations, as verified in the studied *Cystoseira* COI sequences, are considered to be prerequisites for a marker to be considered a suitable DNA barcode [83]. Thus, these results suggest that the COI is useful to differentiate *Cystoseira* taxa, and in particular *C*. *mediterranea* from *C*. *tamariscifolia* and *C*. *amentacea*.

Even though other mitochondrial markers have been used to analyse the phylogeny of brown algae, the results of this study are consistent with those of Silberfeld et al. [42], and also with those of Draisma et al. [34] and Rožić et al. [39] who studied 23S, mt-spacer and/or psbA loci. In certain cases, individual markers were shown not to be sufficiently informative to infer relationships between species [34, 39]. Therefore, multi-gene datasets have been used to improve phylogenetic resolution [34, 41, 42, 84–86]. The phylogenetic trees obtained from the combined datasets used in this work (only *Cystoseira* samples, and *Cystoseira* together with other Sargassaceae) were congruent with previous phylogenies of Fucales [34, 39, 87–89]. Even though COI, 23S and mt-spacer markers resolved several taxa, the polyphyletic nature of the genus *Cystoseira* is a clear obstacle for further taxonomic resolution. As shown by Rousseau and de Reviers [41] and Draisma et al. [34], the Sargassaceae family includes a few polyphyletic genera, such as *Cystoseira*, *Sargassum* and *Bifurcaria*, and consequently there is still much to define within this family.

In spite of the current limitations, the comparative phylogenies of several Sargassaceae with three genetic markers and the divergence analysis enabled the authors to assign previously unidentified samples (*Cystoseira* sp. 1, *Cystoseira* sp. 2, *Cystoseira* sp. MP1, *Cystoseira* sp. MP14, *Cystoseira* sp. MP2, *Cystoseira* sp. MP31) to their respective taxa at the species level. In particular, based on the phylogenetic data gathered in this work, we were able to classify the following samples: *Cystoseira* sp. 1 as *C*. *brachycarpa* (Cystoseira-I); *Cystoseira* sp. 2 as *C*. *montagnei*, *Cystoseira* sp. MP14 as *C*. *abies-marina* (Cystoseira-II); and *Cystoseira* sp. MP31 as *C*. *humilis* var. *humilis*, *Cystoseira* sp. MP1 and *Cystoseira* sp. MP2 as *C*. *humilis* (Cystoseira-III).

Considering its chemical composition, the genus *Cystoseira* has a wide variety of secondary metabolites associated with specific pharmacological properties [19]. For example, it has been shown that *C*. *barbata*, *C*. *compressa*, *C*. *crinita*, *C*. *nodicaulis*, *C*. *tamariscifolia*, and *C*. *usneoides* contain bioactive biochemicals with antioxidant, cholinesterase inhibition, anti-diabetic, anti-cancer, anti-obesity, and anti-inflammatory properties. Interestingly, a few of these activities have been linked to the occurrence of fucosterol [90]. The discovery of bioactive natural products requires an unequivocal identification of the biological specimen, specific sampling, and dereplication strategies in order to efficiently survey the chemical diversity of the target organisms [18, 29, 91]. Because of the ecological, economical and biomedical relevance of *Cystoseira*, further studies on the taxonomic assignment of specimens belonging to this taxon are clearly needed.

## Conclusions

Comprising 22 different *Cystoseira* species and infra-generic taxa currently accepted, this work shows that the identification of the *Cystoseira* specimens using molecular markers is more effective when only closely related individuals are chosen in order to minimize the number and extension of gaps in the alignment of highly variable regions. The combined use of genetic markers with more conserved evolutionary signals (e.g., COI) with highly variable loci such as the mt-spacer allowed for a better resolution of the taxonomic relationships within this group of macroalgae. Given the high variability of the mt-spacer, this marker can be used in combination with COI to distinguish the majority of the *Cystoseira* taxa, resolving the phylogeny of several species of different groups, namely *C*. *barbata* and *C*. *baccata* (Cystoseira-II), and *C*. *foeniculacea* (Cystoseira-III). In addition, the mt-spacer allowed the identification of several distinct haplotypes, particularly in the highly diverse subgroup IIA of the Cystoseira-II clade. Despite some exceptions, our results and the chemotaxonomic classifications suggest that the relationships defined by the phylogenetic, chemical and morphological classifications may be combined and should not be promptly discarded. Moreover, our results indicate that European *Cystoseira*, as currently defined, should be split into three separate genera, to reflect their distinct evolutionary histories, relationships with other genera, and genetic divergence. However, the authors think, at this moment, it is premature to put forward a reclassification of these genera because a perfect match between phylogenetics and morphological traits has not yet been achieved. Before such undertaking can be made, additional species and populations that have not been included in the present study should be sampled and analysed, preferably by means of other (e.g., nuclear) markers. For example, *C*. *abies-marina* fronds seem to be more genetically homogeneous than the specimens identified as *C*. *tamariscifolia*, even though they came from different locations. The higher variability of the latter specimens was most probably the reason why we were unable to distinguish *C*. *tamariscifolia* from *C*. *amentacea* using the markers under study. Hence, these results strongly suggest that a combined effort should be carried out to further elucidate the taxonomy, chemical profiles, anatomical traits and phylogeny of these three groups of *Cystoseira*, using, for example, a whole-genome approach that could identify other markers potentially useful for *Cystoseira* barcoding as well as further resolve the genetic relationships within this genus. Whole-genome markers could also be useful to investigate functional and adaptation traits specific of these algae in the Atlantic-Mediterranean regions and define conservation strategies.

## Supporting information

S1 Table Information on the sequences included in this study—species, geographical origin, voucher, GenBank accession numbers and haplotypes. (PDF)

S2 Table Evolutionary divergence between COI *Cystoseira* sequences. (PDF)

S3 Table Evolutionary divergence between 23S *Cystoseira* sequences. (PDF)

S4 Table Evolutionary divergence between mt-spacer *Cystoseira* sequences. (PDF)

S5 Table Morphological traits identified by other authors for the different *Cystoseira* phylogenetic groups included in this study. (PDF)

S1 Fig Bayesian phylogenetic tree obtained with MrBayes and based on concatenated COI 23S-mt-spacer sequences of the samples from the Sargassaceae family. Values on the branches represent Bayesian posterior probabilities ≥ 90%. Information of the sequences included in this tree are indicated in S1 Table.
(PNG)

S2 Fig Bayesian phylogenetic tree obtained with MrBayes and based on concatenated COI-23S-mt-spacer sequences of the samples from *Cystoseira* genus. Values on the branches represent Bayesian posterior probabilities ≥ 90%. Information of the sequences included in this tree are indicated in S1 Table.
(PNG)

S3 Fig Maximum likelihood phylogenetic tree obtained with RAxML and based on the COI sequences of the samples from *Cystoseira* genus. Values on the branches represent maximum likelihood bootstrap support values ≥ 75 on the left, and Bayesian posterior probabilities ≥ 90% on the right. Information of the sequences included in this tree are indicated in S1 Table.
(PNG)

S4 Fig Bayesian phylogenetic tree obtained with MrBayes and based on the COI sequences of the samples from *Cystoseira* genus. Values on the branches represent Bayesian posterior probabilities ≥ 90%. Information of the sequences included in this tree are indicated in S1 Table.
(PNG)

S5 Fig Maximum likelihood phylogenetic tree obtained with RAxML and based on the 23S sequences of the samples from the *Cystoseira* genus. Values on the branches represent maximum likelihood bootstrap support values ≥ 75 on the left, and Bayesian posterior probabilities ≥ 90% on the right. Information of the sequences included in this tree are indicated in S1 Table.
(PNG)

S6 Fig Bayesian phylogenetic tree obtained with MrBayes and based on the 23S sequences of the samples from *Cystoseira* genus. Values on the branches represet Bayesian posterior probabilities ≥ 90%. Information of the sequences included in this tree are indicated in S1 Table.
(PNG)

S7 Fig Maximum likelihood phylogenetic tree obtained with RAxML and based on the mt-spacer sequences of the samples from *Cystoseira* genus. Values on the branches represent maximum likelihood bootstrap support values ≥ 75 on the left, and Bayesian posterior probabilities ≥ 90% on the right. Information of the sequences included in this tree are indicated in S1 Table.
(PNG)

S8 Fig Bayesian phylogenetic tree obtained with MrBayes and based on the mt-spacer sequences of the samples from *Cystoseira* genus. Values on the branches represent Bayesian posterior probabilities ≥ 90%. Information of the sequences included in this tree are indicated in S1 Table.
(PNG)

S9 Fig Median-Joining networks of Cystoseira-I mt-spacer, 23S and COI haplotypes. **Pie charts are proportional to haplotype frequencies.** Theoretical median vectors are represented by black dots. Colors represent the different *Cystoseira* species as described in the legend.
(PNG)

S10 Fig Median-Joining networks of Cystoseira-II mt-spacer, 23S and COI haplotypes. **Pie charts are proportional to haplotype frequencies.** Theoretical median vectors are represented by black dots. Colors represent the different *Cystoseira* species as described in the legend.
(PNG)

S11 Fig Median-Joining networks of Cystoseira-III mt-spacer, 23S and COI haplotypes. **Pie charts are proportional to haplotype frequencies.** Theoretical median vectors are represented by black dots. Colors represent the different *Cystoseira* species as described in the legend.
(PNG)

## References

[1] Ballesteros E, Torras X, Pinedo S, Garcia M, Mangialajo L, de Torres M. A new methodology based on littoral community cartography dominated by macroalgae for the implementation of the European Water Framework Directive. Mar Pollut Bull. 2007;55: 172–180. 10.1016/j.marpolbul.2006.08.038 17045303

[2] Thibaut T, Blanfuné A, Markovic L, Verlaque M, Boudouresque CF, Perret-Boudouresque M, et al Unexpected abundance and long-term relative stability of the brown alga *Cystoseira amentacea*, hitherto regarded as a threatened species, in the north-western Mediterranean Sea. Mar Pollut Bull. 2014;89: 305–323. 10.1016/j.marpolbul.2014.09.043 25440190

[3] Bermejo R, de la Fuente G, Vergara JJ, Hernández I. Application of the CARLIT index along a biogeographical gradient in the Alboran Sea (European Coast). Mar Pollut Bull 2013;72(1): 107–118. 10.1016/j.marpolbul.2013.04.011 23673205

[4] Bermejo R, Ramírez-Romero E, Vergara JJ, Hernándeza I. Spatial patterns of macrophyte composition and landscape along the rocky shores of the Mediterranean-Atlantic transition region (northern Alboran Sea). Estuar Coast Shelf Sci. 2015;155: 17–28. 10.1016/j.ecss.2015.01.009

[5] Bellan G, Bellan-Santini D. Influence de la pollution sur les peuplements marins de la region de Marseille In: Ruivo M, editor. Marine Pollution and sea life. FAO publication. Fishing News (Books), London; 1972 pp. 396–401.

[6] Bulleri F, Benedetti–Cecchi L, Acunto S, Cinelli F, Hawkins SJ. The influence of canopy algae on vertical patterns of distribution of low-shore assemblages on rocky coasts in the northwest Mediterranean. J Exp Mar Biol Ecol. 2002;267: 89–106. 10.1007/s004420051028

[7] Cheminée A, Sala E, Pastor J, Bodilis P, Thiriet P, Mangialajo L, et al Nursery value of *Cystoseira* forests for Mediterranean rocky reef fishes. J Exp MarBiol Ecol. 2013;442: 70–79. 10.1016/j.jembe.2013.02.003

[8] Bermejo R, de la Fuente G, Ramírez-Romero E, Vergara JJ, Hernández I. Spatial variability and response to anthropogenic pressures of assemblages dominated by a habitat forming seaweed sensitive to pollution (northern coast of Alboran Sea). Mar Pollut Bull. 2016;105: 255–264. 10.1016/j.marpolbul.2016.02.017 26892204

[9] Thibaut T, Pinedo S, Torras X, Ballesteros E. Long-term decline of the populations of Fucales (*Cystoseira* spp. and Sargassum spp.) in the Alberes coast (France, North-western Mediterranean). Mar Pollut Bull. 2005;50: 1472–1489. 10.1016/j.marpolbul.2005.06.014 16026805

[10] Mineur F, Arenasc F, Assisd J, Daviese AJ, Engelend AH, Fernandesd F, et al European seaweeds under pressure: Consequences for communities and ecosystem functioning. J Sea Res. 2015;98: 91–108. 10.1016/j.seares.2014.11.004

[11] Thibaut T, Blanfuné A, Boudouresque CF, Verlaque M. Decline and local extinction of Fucales in the French Riviera: the harbinger of future extinctions? Mediterr Mar Sci. 2015;16: 206–224. 10.12681/mms.1032

[12] Airoldi L, Beck MW. Loss, status and trends for coastal marine habitats of Europe. Oceanogr Mar Biol Annu. Rev. 2007;45: 345–405. 10.1201/9781420050943.ch7

[13] Mangialajo L, Chiantore M, Cattaneo-Vietti R. Loss of fucoid algae along a gradient of urbanisation, and structure of benthic assemblages. Mar Ecol Prog Ser. 2008;358: 63–74. 10.3354/meps07400

[14] Sales M, Cebrian E, Tomas F, Ballesteros E. Pollution impacts and recovery potential in three species of the genus *Cystoseira* (Fucales, Heterokontophyta). Estuar Coast Shelf Sci. 2011;92: 347–357. 10.1016/j.ecss.2011.01.008

[15] Lejeusne C, Chevaldonne Ṕ, Pergent-Martini C, Boudouresque CF, Perez T. Climate change effects on a miniature ocean: the highly diverse, highly impacted Mediterranean Sea. Trends Ecol Evol. 2010, 25: 250–60. 10.1016/j.tree.2009.10.009 19959253

[16] Assis J, Berecibar E, Claro B, Alberto F, Reed D, Raimondi P, Serrão EA. Major shifts at the range edge of marine forests: the combined effects of climate changes and limited dispersal. Sci Rep. 2017,7: 44348 10.1038/srep44348 28276501 PMC5343584

[17] Gianni F, Bartolini F, Airoldi L, Ballesteros E, Francour P, Guidetti P, et al Conservation and restoration of marine forests in the Mediterranean Sea and the potential role of Marine Protected Areas, AIOL, 2013;4: 83–101, 10.1080/19475721.2013.845604

[18] Amico V. Marine brown algae of family Cystoseiraceae: chemistry and chemotaxonomy. Phytochem. 1995;39: 1257–1279. 10.1016/0031-9422(95)00199-H.

[19] Bruno de Sousa C, Gangadhar KN, Macridachis J, Pavão M, Morais TR, Campino L, Varela J, Lago JHG. *Cystoseira* algae (Fucaceae): update on their chemical entities and biological activities. Tetrahedron: Asymmetry. 2017;28: 1486–1505.

[20] Calvo MA, Cabafies EJ, Abarca L. Antifungal activity of some mediterranean algae. Mycopathologia. 1986;93: 61–63. 10.1007/BF00437016 3960102

[21] Spavieri J, Allmendinger A, Kaiser M, Casey R, Hingley-Wilson S, Lalvani A, et al Antimycobacterial, antiprotozoal and cytotoxic potential of twenty-one brown algae (Phaeophyceae) from British and Irish waters. Phytother Res. 2010;24: 1724–1729. 10.1002/ptr.3208 20564461

[22] Mhadhebi L, Laroche-Clary A, Robert J, Bouraoui A. Anti-inflammatory, antiproliferative and antioxidant activities of organic extracts from the Mediterranean seaweed, *Cystoseira crinita*. African J Biotechnol. 2011;10: 16682–16690. 10.5897/AJB11.218 22115493

[23] Pujol C, Ray S, Ray B, Damonte E. Antiviral activity against dengue virus of diverse classes of algal sulfated polysaccharides. Int J Biol Macromol. 2012;51: 412–6. 10.1016/j.ijbiomac.2012.05.028 22652218

[24] de los Reyes C, Ortega MJ, Zbakh H, Motilva V, Zubía E. *Cystoseira usneoides*: A Brown Alga Rich in Antioxidant and Anti-inflammatory Meroditerpenoids. J Nat Prod. 2016;79: 395–405. 10.1021/acs.jnatprod.5b01067 26859694

[25] Vizetto-Duarte C, Custódio L, Acosta G, Lago JHG, Morais TR, Bruno de Sousa C, et al Can macroalgae provide promising anti-tumoral compounds? A closer look at *Cystoseira tamariscifolia* as a source for antioxidant and anti-hepatocarcinoma compounds. PeerJ. 2016;4: e1704 10.7717/peerj.1704 26925328 PMC4768693

[26] Bruno de Sousa C, Gangadhar KN, Morais TR, Conserva GA, Vizetto-Duarte C, Pereira H, et al Antileishmanial activity of meroditerpenoids from the macroalgae *Cystoseira baccata*. Exp Parasitol. 2017;174: 1–9. 10.1016/j.exppara.2017.01.002 28126391

[27] Amico V, Giaccone G, Colombo P, Colonna P., Mannino AM, Randazzo R. Un nuovo approccio allo studio della sistematica del genere *Cystoseira* C. Agardh (Phaeophyta, Fucales). Boll Accad Gioenia Sci Nat Catania. 1985;18: 887–986.

[28] Valls R, Piovetti L, Banaigs B, Praud A. Secondary metabolites from morocco brown algae of the genus *Cystoseira*. Phytochem. 1993;32(4): 961–966. 10.1016/0031-9422(93)85236-K

[29] Leal MC, Hilário A, Munro MHG, Blunt JW, Calado R. Natural products discovery needs improved taxonomic and geographic information. Nat Prod Rep. 2016;33: 747–750. 10.1039/c5np00130g . 26892141

[30] Gómez-Garreta A, Ribera MA, Barceló MC, Rull-Lluch J. Mapas de distribución de algas marinas de la Península Ibérica e Islas Baleares. V. *Cystoseira* C. Agardh: Grupos *C*. *ericaecifolia* y *C*. *crinito-selaginoides*. Bot Complut. 1994;19: 109–118. 10.5209/BOCM.7331

[31] Ballesteros E, Pinedo S. Los bosques de algas pardas y rojas In: Luque del Villar AA, Templado J. editors. Praderas y bosques marinos de Andalucía. Consejería de Medio Ambiente, Junta de Andalucía Sevilla; 2004 pp. 199–222.

[32] Roberts M. Active speciation in the taxonomy of the genus *Cystoseira* C. Ag In: Irvine DEG. & Price JH. Editors. Modern approaches to the taxonomy of red and brown algae. Academic Press, London; 1978 pp. 399–422.

[33] Comarci M, Furnani G, Giaccone G, Scamacca B, Serio D. Observations taxonomiques et biogeographiques sur quelques especes du genre *Cystoseira* C. Agardh. Bull Inst Oceanogr. 1992;9: 21–36.

[34] Draisma S, Ballesteros E, Rousseau F, Thibaut T. DNA Sequence data demonstrate the polyphyly of the genus *Cystoseira* and other Sargassaceae genera (Phaeophyceae). J Phycol. 2010;46: 1329–1345. 10.1111/j.1529-8817.2010.00891.x

[35] Piattelli M. Chemistry and taxonomy of Sicilian *Cystoseira* species. New J Chem. 1990; 14: 777–782.

[36] Valls R, Piovetti L. The chemistry of the Cystoseiraceae (Fucales: Pheophyceae): chemotaxonomic relationships. Biochem Syst Ecol. 1995;23: 723–745. 10.1016/0305-1978(95)00068-2.

[37] Jégou C, Culioli G, Kervarec N, Simon G, Stiger-Pouvreau V. LC/ESI-MS^n^ and ^1^H HR-MAS NMR analytical methods as useful taxonomical tools within the genus *Cystoseira* C. Agardh (Fucales; Phaeophyceae). Talanta. 2010;83: 613–622. 10.1016/j.talanta.2010.10.003 21111182

[38] Gómez-Garreta A, Barceló-Martí MC, Ribera-Siguan MA, Rull-Lluch J. Cystoseira In: Gómez-Garreta A. editor. Flora phycologica iberica. Vol. 1—Fucales. 1^st^ ed; Murcia, Universidad de Murcia; 2001 pp. 99–166. 10.1017/S0967026202003530

[39] Rožić S, Puizina J, Šamanić I, Žuljević A, Antolić B. Molecular identification of the brown algae, *Cystoseira* spp. (Phaeophyceae, Fucales) from the Adriatic Sea—preliminary results. Acta Adriatica. 2012;53: 447–456. ISSN 0001-5113.

[40] Susini M-L. Statut et biologie de Cystoseira amentacea var. stricta. PhD Thesis, The Université de Nice–Sophia Antipolis–UFR Sciences. 2006. 237pp. Available from: ftp://nephi.unice.fr/users/ecomers/2006/2006%20Susini%20Thèse.pdf

[41] Rousseau F, de Reviers B. Phylogenetic relationships within the Fucales (Phaeophyceae) based on combined partial SSU + LSU rDNA sequence data. Eur J Phycol. 1999;34(1): 53–64. 10.1080/09670269910001736082

[42] Silberfeld T, Leigh JW, Verbruggen H, Cruaud C, de Reviers B, Rousseau F. A multi-locus time-calibrated phylogeny of the brown algae (Heterokonta, Ochrophyta, Phaeophyceae): Investigating the evolutionary nature of the ‘‘brown algal crown radiation”. Mol Phylogenet Evol. 2010;56: 659–674. 10.1016/j.ympev.2010.04.020 . 20412862

[43] García-Fernández A, Bárbara I. Studies of *Cystoseira* assemblages in Northern Atlantic Iberia. Anales del Jardín Botánico de Madrid. 2016;73(1): e035 2016. 10.3989/ajbm.2403

[44] Guiry MD, Guiry GM. AlgaeBase. World-wide electronic publication, National University of Ireland, Galway 2017 [cited 2018 Mar 5]. Available from: http://www.algaebase.org.

[45] Arif IA., Khan HA. Molecular markers for biodiversity analysis of wildlife animals: a brief review. Anim Biodivers Conserv. 2009;32: 9–17. ISSN: 1578–665X

[46] Hebert PDN, Cywinska A, Ball SL, Waard JR. Biological identifications through DNA barcodes. Proc R Soc Lond, B. 2003;270: 31–321. 10.1098/rspb.2002.2218 . 12614582 PMC1691236

[47] Aly SM. Reliability of long vs short COI markers in identification of forensically important flies. Croat Med J. 2014;55(1): 19–26. 10.3325/cmj.2014.55.19 24577823 PMC3944415

[48] Sherwood AR., Sauvage T, Kurihara A, Conklin KY, Prestinga GG. A comparative analysis of COI, LSU and UPA marker data for the Hawaiian florideophyte Rhodophyta: Implications for DNA barcoding of red algae. Cryptogamie Algol. 2010;31: 451–465.

[49] Mattio L, Payri C. Assessment of five markers as potential barcodes for identifying *Sargassum* subgenus *Sargassum* species (Phaeophyceae, Fucales). Cryptogamie Algol. 2010;31(4): 467–485.ISSN: 0181-1568.

[50] McDevit DC, Saunders GW. On the utility of DNA barcoding for species differentiation among brown macroalgae (Phaeophyceae) including a novel extraction protocol. Phycol Res. 2009;57: 131–141. 10.1111/j.1440-1835.2009.00530.x

[51] Saunders GW, McDevit DC. DNA barcoding unmasks overlooked diversity improving knowledge on the composition and origins of the Churchill algal flora. BMC Ecol. 2013;13: 9 10.1186/1472-6785-13-9 23497234 PMC3606624

[52] Cormaci M, Furnari G, Catra M, Alongi G, Giaccone G. Flora marina bentonica del Mediterraneo: Phaeophyceae. Boll Accad Gioenia Sci Nat Catania. 2012; 45: 1–508.

[53] Benson DA; Cavanaugh M, Clark K, Karsch-Mizrachi I, Lipman DJ, Ostell J, Sayers IW. GenBank. Nucleic Acids Research. 2013;41: D36–D42. 10.1093/nar/gks1195 23193287 PMC3531190

[54] Doyle JJ, Doyle JL. A rapid DNA isolation for small quantities of fresh leaf tissue. Phytochem Bull. 1987;19: 11–15.

[55] Lane CE, Lindstrom S, Saunders GW. A molecular assessment of northeast Pacific Alaria species (Laminariales, Phaeophyceae) with reference to the utility of DNA barcoding. Mol Phylogenet Evol. 2007;44: 634–648. 10.1016/j.ympev.2007.03.016 17544704

[56] Altschul SF, Gish W, Miller W, Myers EW, Lipman DJ. Basic local alignment search tool. J Mol Biol. 1990;215: 403–410. 10.1016/S0022-2836(05)80360-2 2231712

[57] Bininda-Emonds OR. TransAlign: using amino acids to facilitate the multiple alignment of protein-coding DNA sequences. BMC Bioinformatics. 2005;6: 156 10.1186/1471-2105-6-156 15969769 PMC1175081

[58] Higgins DG, Thompson JD, Gibson TJ. Using CLUSTAL for multiple sequence alignments. Methods Enzymol. 1996;266: 383–402. 10.1016/S0076-6879(96)66024-8 8743695

[59] Talavera G, Castresana J. Improvement of phylogenies after removing divergent and ambiguously aligned blocks from protein sequence alignments. Syst Biol. 2007;56: 564–577. 10.1080/10635150701472164 17654362

[60] Dereeper A, Guignon V, Blanc G, Audic S, Buffet S. Chevenet F, Dufayard JF. Guindon S, Lefort V, Lescot M, Claverie JM, Gascuel O. Phylogeny.fr: robust phylogenetic analysis for the non-specialist. Nucleic Acids Res. 2008;36: 465–9. 10.1093/nar/gkn180 18424797 PMC2447785

[61] Gouy M, Guindon S, Gascuel O. SeaView Version 4: A Multiplatform Graphical User Interface for Sequence Alignment and Phylogenetic Tree Building. Mol Biol Evol. 2010;27(2): 221–224. 10.1093/molbev/msp259 19854763

[62] Librado P, Rozas J. DnaSP v5: A software for comprehensive analysis of DNA polymorphism data. Bioinformatics. 2009;25: 1451–1452. 10.1093/bioinformatics/btp187 19346325

[63] Kimura M. A simple method for estimating evolutionary rate of base substitutions through comparative studies of nucleotide sequences. J Mol Evol 1980;16: 111–120. 10.1007/BF01731581 7463489

[64] Tamura K, Peterson D, Peterson N, Stecher G, Nei M, Kumar S. MEGA5: Molecular evolutionary genetics analysis using maximum likelihood, evolutionary distance, and maximum parsimony methods. Mol Biol Evol 2011;28: 2731–2739. 10.1093/molbev/msr121 21546353 PMC3203626

[65] Nylander JAA. MrModeltest 2.0. Program distributed by the author Evolutionary Biology Centre, Uppsala University. Norbyvagen 18 D. SE-752 36, Uppsala, Sweden 2004 [cited 2017 Mar 5]. Available from: https://github.com/nylander/MrModeltest2

[66] Swofford DL. PAUP*. Phylogenetic Analysis Using Parsimony (*and Other Methods). Version 4. Sinauer Associates, Sunderland, Massachusetts 2003 10.1111/j.0014-3820.2002.tb00191.x

[67] Akaike H. A new look at the statistical model identification. IEEE Trans Autom Control. 1974;19: 716–723. 10.1109/TAC.1974.1100705

[68] Stamatakis A. RAxML-VI-HPC: maximum likelihood-based phylogenetic analyses with thousands of taxa and mixed models. Bioinformatics. 2006;22: 2688–90. 10.1093/bioinformatics/btl446 16928733

[69] Huelsenbeck JP, Ronquist F. MrBayes: Bayesian inference of phylogenetic trees. Bioinformatics. 2001;17: 754–5. 11524383 10.1093/bioinformatics/17.8.754

[70] Ronquist F, Huelsenbeck JP. MrBayes 3: Bayesian inference of phylogenetic trees under mixed model. Bioinformatics. 2003;19: 1572–4. 10.1093/bioinformatics/btg180 12912839

[71] Rambaut A, Drummond AJ. FigTree software. 2009. [cited 2017 Mar 5]. Available from: http://tree.bio.ed.ac.uk./software/figtree/.

[72] Bandelt HJ, Forster P, Röhl A. Median-joining networks for inferring intraspecific phylogenies. Mol Biol Evol. 1999;16: 37–48. 10.1093/oxfordjournals.molbev.a026036 10331250

[73] Giaccone G, Bruni A. Le Cystoseire e la vegetazaione sommersa del mediterraneo. Atti Ist. Ven Sci Lett Arti. 1973;131: 59–103.

[74] Saunders GW. Applying DNA barcoding to red macroalgae: a preliminary appraisal holds promise for future applications. Philos Trans R Soc Lond, B. 2005;360: 1879–1888. 10.1098/rstb.2005.1719 16214745 PMC1609223

[75] Kucera A, Saunders GW. Assigning morphological variants of *Fucus* (Fucales, Phaeophyceae) in Canadian waters to recognized species using DNA barcoding. Botany 2008;86: 1065–1079. 10.1139/B08-056

[76] Clayton MN. The adaptive significance of life history characters in selected orders of marine brown macroalgae. Australian J Ecol. 1990;15: 439–452. 10.1111/j.1442-9993.1990.tb01469.x

[77] Guern M. Embryologie de quelques espéces du genre *Cystoseira* Agardh 1821 (Fucales). Vie et Milieu, Serie A Biologie Marine. 1962;13: 649–679.

[78] Coleman MA, Brawley SH. Spatial and temporal variability in dispersal and population genetic structure of a rockpool alga. Mar Ecol Prog Ser. 2005;300: 63–77. 10.3354/meps300063

[79] Susini M–L, Thibaut T, Meinesz A, Forcioli D. A preliminary study of genetic diversity in *Cystoseira amentacea* (C. Agardh) Bory var. *stricta* Montagne (Fucales, Phaeophyceae) using random amplified polymorphic DNA. Phycologia. 2007;46: 605–611. 10.2216/06-100.1

[80] Colombo P, Curcio MF, Giaccone G. Biologia dello sviluppo di un endemismo mediterraneo del genere *Cystoseira—*Phaeophyceae, Fucales: *Cystoseira sedoides* C. Agardh. Naturalista Sicil. 1982;6: 81–93.

[81] Coyer JA, Hoaray G, Oudot-Le Secq M-P, Stam WT, Olsen JL. A mtDNA-based phylogeny of the brown algal genus *Fucus* (Heterokontophyta; Phaeophyta). Mol Phylogenet Evol. 2006;39: 209–222. 10.1016/j.ympev.2006.01.019 16495086

[82] Ballesteros E, Catalán J. Flora y vegetación marina y litoral del Cabo de Gata y el Puerto de Roquetas de Mar (Almería). Primera aproximación. An la Univ Murcia,1981;42: 237–277.

[83] Saunders GW, Kucera H. An evaluation of rbcL, tufA, UPA, LSU and ITS as DNA barcode markers for the marine green macroalgae. Cryptogamie, Algologie, 2010;31: 487–528

[84] Lane CE, Mayes C. A multi-gene molecular investigation of the kelp (laminariales, Phaeophyceae) supports substantial taxonomic reorganization. J Phycol. 2006;42: 493–512. 10.1111/j.1529-8817.2006.00204.x

[85] Vaidya G, Lohman DJ, Meier R. Sequence Matrix: concatenation software for the fast assembly of multi-gene datasets with character set and codon information. Cladistics. 2011; 27: 171–180. 10.1111/j.1096-0031.2010.00329.x 34875773

[86] Lam DW, Verbruggen H, Saunders GW, Vis ML. Multigene phylogeny of the red algal subclass Nemaliophycidae. Mol Phylogenet Evol. 2016;94: 730–736. 10.1016/j.ympev.2015.10.015 26518739

[87] Phillips NE; Smith CM, Morden CW. Testing systematic concepts of *Sargassum* (Fucales, Phaeophyceae) using portions of the rbcLS operon. Phycological Res. 2005;53: 1–10. 10.1111/j.1440-183.2005.00368.x

[88] Cho GY, Rousseau F, de Reviers B, Boo SM. Phylogenetic relationships within the Fucales (Phaeophyceae) assessed by the photosystem I coding *psa*A sequences. Phycologia. 2006;45:512–519. 10.2216/05-48.1

[89] Harvey JBJ, Goff LJ. A reassessment of species boundaries in *Cystoseira* and *Halidrys* (Phaeophyceae, Fucales) along the North American West coast. J Phycol. 2006;42: 707–720. 10.1111/j.1529-8817.2006.00215.x

[90] Andrade PB, Barbosa M, Matos RP, Lopes G.; Vinholes J, Mouga T, Valentão P. Valuable compounds in macroalgae extracts. Food Chem. 2013, 138: 1819–1828. 10.1016/j.foodchem.2012.11.081 23411314

[91] Bucar F, Wube A, Schmid M. Natural product isolation–how to get from biological material to pure compounds. Nat Prod Rep. 2013, 30: 525–45. 10.1039/c3np20106f 23396532

