# Supplementary material for: Improved phylogeny of brown algae Cystoseira (Fucales) from the Atlantic-Mediterranean region based on mitochondrial sequences
Source: PLoS One. 2019 Jan 30;14(1):e0210143. doi: 10.1371/journal.pone.0210143 (PMC6364706; doi:10.1371/journal.pone.0210143)
Supplement: S1 Table — (PDF) [file pone.0210143.s001.pdf]

S1Table. Information of the sequences included in this study - species, geographical origin, voucher, GenBank accession numbers and haplotypes.

**Sample**		**Geographic origin**		**Sample information and sequences GenBank acession n.º**				
**Species**	**Reference**	**Country**	**Location**	**Voucher**	**COI**	**23S**	**mt-spacer**	**Author**
Cystoseira abies-marina (S.G.Gmelin) C.Agardh	C. abies marina 1	Spain	Tenerife, Mesa del Mar	L:0609335	MP3#	FM958377	FM993042	Draisma
Cystoseira abies-marina (S.G.Gmelin) C.Agardh	C. abies marina 2	Spain	Tenerife, Bajamar	L:0609338	MP3#	FM958376	FM993042	Draisma
Cystoseira abies-marina (S.G.Gmelin) C.Agardh	C. abies marina MP19	Spain	Tenerife, Punta del Hidalgo	MD0000400	MF768067	MF767968	-	This study
Cystoseira abies-marina (S.G.Gmelin) C.Agardh	C. abies marina MP26	Spain	Tenerife, Punta del Hidalgo	MD0000522	MF768068	MF767966	-	This study
Cystoseira abies-marina (S.G.Gmelin) C.Agardh	C. abies marina MP27	Spain	Tenerife, Punta del Hidalgo	MD0000557	MF768069	MF767967	MF768019	This study
Cystoseira abies-marina (S.G.Gmelin) C.Agardh	C. abies marina MP29	Portugal	S. Miguel ls, Ponta dos Mosteiros	MD0000746	MF768070	MF767969	MF768020	This study
Cystoseira abies-marina (S.G.Gmelin) C.Agardh	C. abies marina MP3	Spain	Tenerife, Punta del Hidalgo	PG072209	MF768066	-	MF768021	This study
Cystoseira abies-marina (S.G.Gmelin) C.Agardh	C. abies marina MP30	Portugal	S. Miguel ls, Ponta dos Mosteiros	MD0000778	MP3#	MF767970	MF768022	This study
Cystoseira amentacea (C.A-gardh) Bory	C. amentacea	Italy	Sicily, Capo Passero	L:0609436	-	FM958359	FM993021	Draisma
Cystoseira amentacea (C.Agardh) Bory	C. amentacea GV3	Spain	Almería, Guardias Viejas	GV3	MF768051	MF767983	MF768004	This study
Cystoseira amentacea (C.Agardh) Bory	C. amentacea MU1	Spain	Murcia, Cabo de Palos	MU1	MF768052	MF767982	-	This study
Cystoseira amentacea var. stricta Montagne	C. amentacea var. stricta 1	Italy	Sicily, Capo Gallo	L:0609384	-	FM958371	FM993017	Draisma
Cystoseira amentacea var. *stricta* Montagne	C. amentacea var. stricta 2	Italy	Sicily, S. Maria la Scala	L:0609446	-	FM958356	FM993016	Draisma
Cystoseira amentacea var. *stricta* Montagne	C. amentacea var. stricta RB95	Spain	Almería, Las Negras	MBR95	MF768050	-	MF768003	This study
Cystoseira baccata (S.G.G-melin) P.C.Silva	C. baccata 1	Spain	A Coruña	SANT:16322	EU681399"	FM958368	FM993034	Draisma
Cystoseira baccata (S.G.Gmelin) P.C.Silva	C. baccata MB1	Portugal	Viana do Castelo, Areosa	MB1	MF768078	MF767978	MF768024	This study
Cystoseira baccata (S.G.Gmelin) P.C.Silva	C. baccata MB2	Portugal	Caminha, Moledo	MB2	MF768079	MF767979	MF768025	This study
Cystoseira barbata (S.G.Gmelin) P.C.Silva	C. barbata	Spain	Menorca, Moll d'es Miami	L:0609316	-	FM958378	FM993043	Draisma

Sample		Geographic origin		Sample information and sequences GenBank acession n.º				
**Species**	**Reference**	**Country**	**Location**	**Voucher**	**COI**	**23S**	**mt-spacer**	**Author**
Cystoseira barbata f.*aurantia* (Kützing) Giaccone	C. barbata f. aurantia RB87	Spain	Cádiz, Santibañez	MBR87	MF768074	MF767972	MF768028	This study
Cystoseira barbata	C. barbata MB17	Spain	Cádiz, Santibañez	MB17	MF768075	MF767971	MF768027	This study
Cystoseira barbata	C. barbata/susanensis	Italy	Sicily, Marzameni	L:SGAD1638	-	FM958379	FM993044	Draisma
Cystoseira barbatula Kützing emendavit Cormaci, G.-Furnari & Giaccone	C. barbatula	Italy	Sicily, Marzameni	L:0609441	-	FM958365	-	Draisma
Cystoseira brachycarpa var. balearica(Sauvageau) Giaccone[†]	C. brachycarpa var. balearica 1	Spain	Menorca, Cala Viola de Llevant	L:0609251	-	FM958361	-	Draisma
Cystoseira brachycarpa var. *balearica*(Sauvageau) Giaccone[†]	C. brachycarpa var. balearica 2	Spain	Menorca, La Llosa d'en Patro Pere	L:0609308	-	FM958362[*]	FM993025	Draisma
Cystoseira brachycarpa var. *balearica* (Sauvageau) Giaccone[†]	C. brachycarpa var. balearica 3	Italy	Sicily, Capo Milazzo	L:0609404	-	FM958362[*]	FM993027	Draisma
Cystoseira brachycarpa J.Agardh	C. brachycarpa 1	Italy	Sicily, S. Maria la Scala	L:0609414	-	FM958362	FM993026	Draisma
Cystoseira brachycarpa J.Agardh	C. brachycarpa 2	Italy	Aeolian Is, Salina	L:SGAD1633	-	-	FM993028	Draisma
Cystoseira brachycarpa J.Agardh	C. brachycarpa 3	France	Cote Vermeille, Banyuls-sur-Mer	PC:FR194	-	-	FM993023	Draisma
Cystoseira compressa (Esper) Gerloff & Nizamuddin	C. compressa	Spain	Tenerife, Punta del Hidalgo	L:0609343	MP17[#]	FM958355	FM993015	Draisma
Cystoseira compressa (Esper) Gerloff & Nizamuddin	C. compressa MB4	Portugal	Albufeira, Arrifes	MB4	MF768038	MF767959	-	This study
Cystoseira compressa (Esper) Gerloff & Nizamuddin	C. compressa MB6	Portugal	Albufeira, Olhos de Água	MB6	MF768039	MF767960	MF767995	This study
Cystoseira compressa (Esper) Gerloff & Nizamuddin	C. compressa MP17	Spain	Tenerife, Mesa del Mar	MD0000360	MF768040	-	-	This study
Cystoseira compressa (Esper) Gerloff & Nizamuddin	C. compressa RB25	Spain	Girona, Blanes	MBR25	MF768041	MF767962	MF767996	This study
Cystoseira compressa (Esper) Gerloff & Nizamuddin	C. compressa MP4	Spain	Tenerife, Mesa del Mar	PG072363	MF768036	-	-	This study
Cystoseira compressa(Esper) Gerloff & Nizamuddin	C. compressa MP25	Spain	Tenerife, Mesa del Mar	MD0000506	MF768037	MF767961	-	This study
Cystoseira compressa subsp.	C. compressa subsp.. pustulata	Italy	Sicily, Marzameni	L:0609427	-	FM958354	FM993014	Draisma

Sample		Geographic origin		Sample information and sequences GenBank acession n.º				
**Species**	**Reference**	**Country**	**Location**	**Voucher**	**COI**	**23S**	**mt-spacer**	**Author**
pustulata (Ercegovic) Verlaque								
Cystoseira compressa subsp. *pustulata* (Ercegovic) Verlaque	C. compressa subsp. pustulata RB67	Spain	Almería, La isleta del Moro	MBR67	MF768043	-	-	This study
Cystoseira compressa subsp. *pustulata* (Ercegovic) Verlaque	C. compressa subsp. pustulata RB103	Spain	Almería, La isleta del Moro	MBR103	MF768042	MF767957	MF767998	This study
Cystoseira crinita Duby	C. crinita 1	Spain	Menorca, Illots de Tirant	L:0609275	-	FM958363	FM993029	Draisma
Cystoseira crinita Duby	C. crinita 2	Italy	Sicily, Marzameni	L:0609440	-	FM958360	FM993024	Draisma
Cystoseira crinita Duby	C. crinita 3	Spain	Menorca, Cala Mica	L:0609314	-	-	FM993030	Draisma
Cystoseira crinita Duby	C. crinita RB90	France	Antibes, Pointe I´lette	MBR90	-	MF767984	MF768018	This study
Cystoseira elegans Sauvageau	C. elegans	Italy	Sicily, Capo Passero	L:0609444	-	FM958375	FM993038	Draisma
*Cystoseira elegans*Sauvageau	C. elegans RB68	Spain	Almería, El Playazo	MBR68	-	MF767973	MF768029	This study
Cystoseira foeniculacea (Linnaeus) Greville	C. foeniculacea	Spain	Tenerife, Punta del Hidalgo	L:0609350	-	FM958353	FM993013	Draisma
Cystoseira foeniculacea (Linnaeus) Greville	C. foeniculacea MP20	Spain	Tenerife, Mesa del Mar	MD0000403	MF768049	-	MF768001	This study
Cystoseira foeniculacea (Linnaeus) Greville	C. foeniculacea MP22	Spain	Tenerife, Mesa del Mar	MD0000421	MF768048	MF767965	MF768002	This study
Cystoseira funkii Schiffner ex Gerloff & Nizamuddin	C. funkii	Italy	Aeolian Is, Salina	L:0609449	-	FM958357	FM993018	Draisma
Cystoseira granulata	C. granulata	France	Brittany	PC:FR295	-	-	FM993039	Draisma
Cystoseira humilis Kützing	C. humilis MB7	Portugal	Albufeira, Manuel Lourenço	MB7	MF768046	KF525359	-	This study
Cystoseira humilis var. *myriophylloides* (Sauvageau) J.H.Price & D.M.John	C. humilis var. myriophylloides RB22	Spain	Cádiz, El Mirlo	MBR22	MF768047	MF767963	MF767997	This study
Cystoseira mauritanica Sauvageau	C. mauritanica RB18	Spain	Cádiz, El Mirlo	MBR18	MF768047	MF767963	MF767997	This study
Cystoseira mediterranea Sauvageau	C. mediterranea	France	Cote Vermeille, Le Troc	L:0609379	-	FM958371*	FM993022	Draisma
Cystoseira mediterranea Sauvageau	C. mediterranea BL14	Spain	Girona, Blanes	BL14	MF768064	KF525356	MF768006	This study
Cystoseira mediterranea Sauvageau	C. mediterranea BL5	Spain	Girona, Blanes	BL5	MF768065	KF525357	KF525365	This study

Sample		Geographic origin		Sample information and sequences GenBank acession n.º				
**Species**	**Reference**	**Country**	**Location**	**Voucher**	**COI**	**23S**	**mt-spacer**	**Author**
Cystoseira nodicaulis (Withering) M.Roberts	C. nodicaulis 2	France	Brittany, Santec	PC FR289	EU681400	FM958369*	FM993036	Draisma
Cystoseira nodicaulis (Withering) M.Roberts	C. nodicaulis MB14	Spain	A Coruña, Santa Mariña	MB14.2	MF768076	MF767974	MF768030	This study
Cystoseira nodicaulis (Withering) M.Roberts	C. nodicaulis MB18	Spain	A Coruña, Santa Mariña	MB18	MF768077	MF767975	MF768031	This study
Cystoseira sonderi (Kützing) Piccone	C. sonderi 1	Cape Verde	Branco Is	L:CANCAP-VII 9718	-	-	FM993040	Draisma
Cystoseira sonderi (Kützing) Piccone	C. sonderi 2	Cape Verde	São Tiago ls, Tarrafal Bay	L:CANCAPVII 8621	-	-	FM993041	Draisma
Cystoseira sp. C.Agardh	Cystoseira sp. 1	Croatia	Prvic Island	GENT: KRK 005	-	FM958364	FM993031	Draisma
Cystoseira sp. C.Agardh	Cystoseira sp. 2	Spain	Menorca, Illa d'es Porros	L:0609306	-	FM958369	FM993035	Draisma
Cystoseira sp. C.Agardh	Cystoseira sp. MP1	Portugal	Madeira Is, Caniço	PG071164	-	MF767964	MF768000	This study
Cystoseira sp. C.Agardh	Cystoseira sp. MP14	Malta	Xghajra	PG081405	MF768071	-	MF768023	This study
Cystoseira sp. C.Agardh	Cystoseira sp. MP2	Portugal	Madeira Is, Caniço	PG071220	MF768045	-	MF767999	This study
Cystoseira sp. C.Agardh	Cystoseira sp. MP31	Portugal	Graciosa Is, Carapacho	MD0003137	MF768044	MF767958	-	This study
Cystoseira sp. C.Agardh	Cystoseira sp. RB105	Spain	Almería, El Playazo	MBR105	-	-	MF768005	This study
Cystoseira montagnei C.Agardh	C. montagnei	Croatia	Brac Island	-	-	HQ438490	HQ438492	Puizina
Cystoseira montagnei C.Agardh	C. montagnei ALI4	Spain	Alicante, Santa Pola	ALI4	MF768072	MF767977	MF768032	This study
Cystoseira montagnei C.Agardh	C. montagnei RB24	Spain	Almería, La Serena	MBR24	-	-	MF768033	This study
Cystoseira montagnei var. *tenuior (Ercegović) M. Verlaque,A. Blanfuné, C.F. Boudouresque, T. Thibaut & L.N. Sellam comb. nov.*	C. montagneivar. tenuior	Spain	Menorca, Cala Mica	L:0609312	-	FM958374	FM993037	Draisma
Cystoseira squarrosa De No-taris	C. squarrosa	Croatia	Dubrovnik city area	-	-	HQ438491	HQ438494	Puizina
Cystoseira tamariscifolia (Hudson) Papenfuss	C. tamariscifolia 1	Spain	A Coruña	SANT16323	EU681401"	FM958358	FM993019	Draisma
Cystoseira tamariscifolia (Hudson) Papenfuss	C. tamariscifolia 3	Spain	A Coruña	SANT:16325	EU681401"	FM958370	FM993020	Draisma
Cystoseira tamariscifolia (Hudson) Papenfuss	C. tamariscifolia CB5	Spain	Málaga, Calaburras	CB5	MF768053	MF767990	MF768007	This study

Sample		Geographic origin		Sample information and sequences GenBank acession n.º				
**Species**	**Reference**	**Country**	**Location**	**Voucher**	**COI**	**23S**	**mt-spacer**	**Author**
Cystoseira tamariscifolia (Hudson) Papenfuss	C. tamariscifolia HE7	Spain	Granada, Herradura	HE7	MF768054	MF767991	MF768011	This study
Cystoseira tamariscifolia (Hudson) Papenfuss	C. tamariscifolia MB10	Portugal	Albufeira, Olhos de Água	MB10	-	MF767992	MF768009	This study
Cystoseira tamariscifolia (Hudson) Papenfuss	C. tamariscifolia MB11	Portugal	Albufeira, Olhos de Água	MB11	MF768055	MF767993	MF768008	This study
Cystoseira tamariscifolia (Hudson) Papenfuss	C. tamariscifolia MB12	Portugal	Albufeira, Olhos de Água	MB12	MF768056	MF767985	MF768010	This study
Cystoseira tamariscifolia (Hudson) Papenfuss	C. tamariscifolia MB16	Portugal	Albufeira, Olhos de Água	MB16	MF768057	-	MF768013	This study
Cystoseira tamariscifolia (Hudson) Papenfuss	C. tamariscifolia MB19	Portugal	Albufeira, Olhos de Água	MB19	MF768058	MF767994	MF768012	This study
Cystoseira tamariscifolia (Hudson) Papenfuss	C. tamariscifolia MB20	Portugal	Albufeira, Olhos de Água	MB20	MF768059	MF767988	MF768014	This study
Cystoseira tamariscifolia (Hudson) Papenfuss	C. tamariscifolia MB5	Portugal	Albufeira, Manuel Lourenço	MB5	MF768061	-	KF525364	This study
Cystoseira tamariscifolia (Hudson) Papenfuss	C. tamariscifolia MB8	Portugal	Viana do Castelo, Areosa	MB8	MF768060	MF767986	MF768015	This study
Cystoseira tamariscifolia (Hudson) Papenfuss	C. tamariscifolia MB9	Portugal	Albufeira, Manuel Lourenço	MB9	MF768062	MF767989	MF768016	This study
Cystoseira tamariscifolia (Hudson) Papenfuss	C. tamariscifolia TA2	Spain	Cádiz, El Mirlo	TA2	MF768063	MF767987	MF768017	This study
Cystoseira usneoides (Linnaeus) M.Roberts	C. usneoides	Spain	A Coruña	SANT:15803	MB13[#]	FM958367	FM993033	Draisma
Cystoseira usneoides (Linnaeus) M.Roberts	C. usneoides MB13	Portugal	Albufeira, Olhos de Água	MB13	MF768080	-	-	This study
Cystoseira usneoides (Linnaeus) M.Roberts	C. usneoides MB15	Portugal	Albufeira, Manuel Lourenço	MB15	MF768081	KF525360	KF525362	This study
Cystoseira usneoides (Linnaeus) M.Roberts	C. usneoides MB21	Portugal	Albufeira, Olhos de Água	MB21	MF768082	MF767980	MF768034	This study
Cystoseira usneoides (Linnaeus) M.Roberts	C. usneoides MB3	Portugal	Aljezur, Odeceixe	MB3	MF768083	MF767981	MF768035	This study
Cystoseira zosteroides (Turner) C.Agardh	C. zosteroides	Italy	Sicily, S. Maria la Scala	L:0609421	-	FM958366	FM993032	Draisma
Bifurcaria bifurcata R.Ross	Bifurcaria bifurcata 1	France	Brittany, Santec	PC:FR287/FRA0520	EU681394	FM958373	FM992996	Draisma
Bifurcaria bifurcata R.Ross	Bifurcaria bifurcata MB34	Spain	A Coruña, Lires	MB34	MB37	x	x	This study
Bifurcaria bifurcata R.Ross	Bifurcaria bifurcata MB35	Spain	A Coruña	MB35	MB37	-	x	This study

Sample		Geographic origin		Sample information and sequences GenBank acession n.º				
**Species**	**Reference**	**Country**	**Location**	**Voucher**	**COI**	**23S**	**mt-spacer**	**Author**
Bifurcaria bifurcata R.Ross	Bifurcaria bifurcata MB36	Spain	Asturias, Porcia	MB36	MB37	x	MB35	This study
Bifurcaria bifurcata R.Ross	Bifurcaria bifurcata MB37	Spain	Murcia, Lastra	MB37	MB37	-	x	This study
Polycladia heinii (Schiffner) Draisma, Ballesteros, F.Rousseau & T.Thibaut	Polycladia heinii	Oman	Al Ashkharah	GENT:ASH 030	-	FM958335	FM992993	Draisma
Polycladia indica (Thivy & Doshi) Draisma, Ballesteros, F.Rousseau & T.Thibaut	Polycladia indica	Oman	Dhofar, Mirbat	GENT:DHO2 0297	-	FN435994	FM992994	Draisma
Sirophysalis trinodis(Forsskal) Kützing	Sirophysalis trinodis	Indonesia/ Australia	Thousand Is, Semak Daun	L:SGAD0509396/ AD-A95058A	KF285949	FM958348	FM993008	Draisma / Soisup
Stephanocystis geminata (C.Agardh) Draisma, Ballesteros, F.Rousseau & T.Thibaut	Stephanocystis geminata	Canada	British Columbia	GWS004223	FJ409138	-	-	McDevit
Stephanocystis hakodatensis (Yendo) Draisma, Ballesteros, F.Rousseau & T.Thibaut	Stephanocystis hakodatensis	Japan	Hokkaido, Muroran	SAP:086290	-	FM958350	FM993010	Draisma
Stephanocystis setchelli (N.L.Gardner) Draisma, Ballesteros, F.Rousseau & T.Thibaut	Stephanocystis setchelli	USA	California, Anacapa Island	AC2	-	FM958351	FM993011	Draisma
Stephanocystis dioica (N.L.Gardner) Draisma, Ballesteros, F.Rousseau & T.Thibaut	Stephanocystis dioica	USA	California, Catalina Island	CT2	-	FM958352	FM993012	Draisma
Turbinaria ornata (Turner)J.Agardh	Turbinaria ornata 3	Indonesia	Thousand Is, Pulau Sepa	L:SGAD0509269 / -	JF718405	FM958414	FM993083	Draisma/ Yu
Fucus distichus Linnaeus	Fucus distichus 1	Spain	Tenerife, Punta del Hidalgo	AY659916/ CSM007A	EU646709	AY659916	AY659884	Draisma/ Kucera
Fucus serratus Linnaeus	Fucus serratus 3	- / Canada	- / Nova Scotia	AY659920/ DM05-014	EU646717	AY659920	AY659875	Draisma/ Kucera
Fucus spiralis Linnaeus	Fucus spiralis 1	- / Canada	- / Nova Scotia	AY659921/ CSM009A	EU646738	AY659921	AY659907	Draisma/ Kucera
Fucus vesiculosus Linnaeus	Fucus vesiculosus 5	- / Canada	- / Nova Scotia	AY494079	AY494079	AY494079	AY494079	Draisma/ Kucera

†Currently considered as synonym of *C. brachycarpa* J.Agardh * Sequences identical to, as indicated by Draisma; # Sequences assumed to be identical to the sequence given because available sequences from other individuals did not show any variation; ¨ Sequences assumed to be identical to other individual from the same specie available at GenBank database.
