# Supplementary material for: Improved phylogeny of brown algae Cystoseira (Fucales) from the Atlantic-Mediterranean region based on mitochondrial sequences
Source: PLoS One. 2019 Jan 30;14(1):e0210143. doi: 10.1371/journal.pone.0210143 (PMC6364706; doi:10.1371/journal.pone.0210143)
Supplement: S2 Table — (PDF) [file pone.0210143.s002.pdf]

S2 Table. Evolutionary divergence between COI *Cystoseira* sequences.

A.

**Group I species**	*C. amentacea* var. *stricta**	*C. amentacea*	*C. tamariscifolia*	*C. mediterranea*
C. amentacea var. *stricta**	*			
C. amentacea	0.3	0.3		
C. tamariscifolia	0.3 - 0.5	0.0 - 0.2	0.0 - 0.2	
C. mediterranea	0.9	0.9	0.9 – 1.1	0.0

B.

**Group II species**	*C. abies-marina*	*Cystoseira sp.* MP14*	*C. montagnei**	*C. mauritanica**	*C. barbata* f. *aurantia**	*C. barbata**	*C. nodicaulis*	*C. baccata*	*C. usneoides*
C. abies-marina	0.0								
*Cystoseira sp. MP14**	0.0	*							
*C. montagnei**	5.6	5.6	*						
*C. mauritanica**	5.4	5.4	0.3	*					
C. barbata f. *aurantia**	5.4	5.4	0.3	0.0	*				
*C. barbata**	5.8	5.8	0.6	0.3	0.3	*			
C. nodicaulis	5.8	5.8	0.6	0.3	0.3	0.0	5.6		
C. baccata	6.3 – 6.8	6.3 – 6.8	3.0 – 3.4	3.0 – 3.4	3.0 – 3.4	3.3 – 3.8	3.3 – 3.8	0.0 – 0.5	
C. usneoides	5.9	5.9	2.3	2.0	2.0	2.3	2.3	1.7 – 2.2	0.0

C.

**Group III species**	*C. compressa*	*C. compressa subsp. pustulata*	*Cystoseira sp.* MP31*	*Cystoseira* sp. MP2*	*C. humilis**	*C. humilis* var. *myriophylloides**	*C. foeniculacea*
C. compressa	0.0 - 0.6						
C. compressa subsp. pustulata	0.9 – 1.0	0.0					
Cystoseira sp. MP31*	1.0	0.0	*				
Cystoseira sp. MP2*	0.6	0.6	0.6	*			
*C. humilis**	0.6	0.6	0.6	0.0	*		
C. humilis var. *myriophylloides**	0.6	0.6	0.6	0.0	0.0	*	
C. foeniculacea	4.2 – 4.4	4.2 – 4.3	4.2 – 4.4	4.2 – 4.4	4.2 – 4.4	4.2 – 4.4	0.0

*Species represented by only one specimen
