# Supplementary material for: Improved phylogeny of brown algae Cystoseira (Fucales) from the Atlantic-Mediterranean region based on mitochondrial sequences
Source: PLoS One. 2019 Jan 30;14(1):e0210143. doi: 10.1371/journal.pone.0210143 (PMC6364706; doi:10.1371/journal.pone.0210143)
Supplement: S3 Table — (PDF) [file pone.0210143.s003.pdf]

S3 Table . Evolutionary divergence between 23S *Cystoseira* sequences.

A.

**Group I species**	*C. zosteroides**	*C. amentacea*	*C. amentacea* var. *stricta*	*C. funkii**	*C. mediterranea*	*Cystoseira* sp. 1*	*C. brachycarpa**	*C. brachycarpa var. balearica*	*C. crinita*	*C. barbatula**	*C. tamariscifolia*
*C. zosteroides**	*										
C. amentacea	1.9 – 2.4	0.5 - 2.2									
C. amentacea var. *stricta*	1.6 – 2.4	0.5 - 1.3	0.8								
*C. funkii**	2.6	1.0 - 2.0	0.8 - 1.0	*							
C. mediterranea	1.6	0.5 - 1.9	0.0 - 0.8	1.0	0.0						
Cystoseira sp. 1*	2.6	3.7 - 4.3	3.2 - 3.5	4.3	3.2	*					
*C. brachycarpa**	3.2	3.5 - 4.3	3.7	3.5	3.7	1.0	*				
C. brachycarpa var. *balearica*	2.9 – 3.2	3.4 - 4.3	3.2 - 3.7	3.5 - 3.7	3.5 - 3.5	0.8 - 1.0	0.0 - 0.3	0.0 - 0.3			
C. crinita	2.4 – 2.9	3.5 - 4.3	2.9 - 3.7	3.5 - 4.0	2.9 - 3.5	0.3 - 0.8	0.3 - 0.8	0.0 - 0.8	0.3 - 0.8		
*C. barbatula**	3.2	4.3 - 4.9	3.7 - 4.3	4.9	3.7	0.8	1.6	1.3 - 1.6	0.8 - 1.6	*	
C. tamariscifolia	1.7 – 2.4	0.0 - 2.3	0.3 - 1.3	1.0 - 1.6	0.3 - 1.0	3.4 - 4.3	3.7 - 4.3	3.5 - 4.3	3.4 - 4.3	4.3 - 4.9	0.0 - 0.6

B.

**Group II species**	*C. abies-marina*	*C. barbata*	*C. barbata* f. *aurantia**	*C. elegans*	*C. nodicaulis*	*C. mauritanica**	*C. montagnei* var. *tenuior**	*C. montagnei*	Cystoseira sp. 2*	*C. squarrosa**	*C. baccata*	*C. usneoides*
C. abies-marina	0.0											
C. barbata	3.2	0.0 - 1.6										
C. barbata f. *aurantia**	3.2	0.0 - 1.6	*									
C. elegans	3.0	0.5 - 1.9	0.5 - 1.1	1.3								
C. nodicaulis	3.2	0.0 - 1.6	0.0	0.5 - 1.1	0.0							
*C. mauritanica**	3.2	0.0 - 1.6	0.0	0.5 - 1.1	0.0	*						
C. montagnei var. *tenuior**	3.2	0.0 - 1.6	0.0	0.5 - 1.1	0.0	0.0	*					
C. montagnei	3.0-3.2	0.0 - 1.9	0.0 - 1.1	0.0 - 1.3	0.0 - 1.1	0.0 - 1.1	0.0 - 1.1	1.1				
Cystoseira sp. 2*	3.2	0.0 - 1.6	0.0	0.5 - 1.1	0.0	0.0	0.0	0.0 - 1.1	*			
*C. squarrosa**	3.0	1.1 - 1.9	1.1	0.0 - 1.3	1.1	1.1	1.1	0.0 - 1.1	1.1	*		
C. baccata	4.3-4.6	2.1 - 3.1	2.1 - 2.3	2.1 - 2.6	2.1 - 2.3	2.1 - 2.3	2.1 - 2.3	2.1 - 2.3	2.1 - 2.3	2.1 - 2.3	0.0	
C. usneoides	1.3	1.9 - 2.9	1.9	1.9 - 2.1	1.9	1.9	1.9	1.9	1.9	1.9	1.0 - 1.1	0.0

C.

**Group III species**	*C. compressa subsp. pustulata*	*Cystoseira* sp. MP31*	*C. compressa*	*C. humilis var. myriophylloides**	*C. humilis**	*Cystoseira* sp. MP1*	*C. foeniculacea*
C. compressa subsp. *pustulata*	0.0						
Cystoseira sp. MP31*	0.0	*					
C. compressa	0.8 - 1.0	0.8 - 1.0	0.0 - 0.3				
C. humilis var. *myriophylloides**	0.5	0.5	0.3 - 0.5	*			
*C. humilis**	0.5	0.5	0.3 - 0.5	0.0	*		
Cystoseira sp. MP1*	0.6	0.6	0.3 - 0.3	0.0	0.0	*	
C. foeniculacea	2.1	2.1	1.8 - 2.1	1.6	1.6	1.7	0.0

* Species represented by only one specimen
