# Supplementary material for: Improved phylogeny of brown algae Cystoseira (Fucales) from the Atlantic-Mediterranean region based on mitochondrial sequences
Source: PLoS One. 2019 Jan 30;14(1):e0210143. doi: 10.1371/journal.pone.0210143 (PMC6364706; doi:10.1371/journal.pone.0210143)
Supplement: S4 Table — (PDF) [file pone.0210143.s004.pdf]

S4 Table. Evolutionary divergence between mt-spacer _Cystoseira_ sequences.

A.

**Group I species**	_C. amentacea_ var. _stricta_	_C. amentacea_	_C. mediterranea_	_C. tamariscifolia_	_C. funkii_*	_Cystoseira_ sp, RB105 *	_C. brachycarpa_ var. _balearica_	_C. brachycarpa_	_C. crinita_	_Cystoseira_ sp. 1*	_C. zosteroides_*
C. amentacea var. _stricta_	1.1 - 2.6										
C. amentacea	0.7 - 4.4	0.4									
C. mediterranea	0.4 - 3.0	1.1 - 1.7	0.0								
C. tamariscifolia	0.4 - 3.9	0.0 - 3.0	0.7 - 1.4	0.0 - 2.6							
_C. funkii_*	1.1 - 3.4	1.8 - 2.6	0.7	1.5 - 2.2	*						
Cystoseira sp.RB105 *	0.4 - 3.0	1.1 - 1.7	0.0	0.7 - 1.5	0.7	*					
C. brachycarpa var. _balearica_	5.6 - 7.0	6.0 - 7.6	5.2 - 5.7	6.0 - 6.8	4.9 - 5.4	5.3 - 5.7	2.9				
C. brachycarpa	5.7 - 7.7	7.6 - 8.5	5.2 - 6.0	6.0 - 7.4	4.9 - 6.1	5.3 - 6.0	0.0 - 2.9	0.4 - 2.5			
C. crinita	1.1 - 9.0	1.8 - 9.5	0.7 - 6.8	1.4 - 8.3	0.0 - 6.5	0.7 - 6.8	2.1 - 3.3	2.1 - 6.8	0.3 - 7.6		
Cystoseira sp. 1*	6.4 - 7.3	6.8 - 8.5	6.0	6.8 - 7.4	6.1	6.0	1.8 - 3.2	1.4 - 3.2	3.5 - 6.8	*	
_C. zosteroides_*	7.3 - 8.7	7.7 - 9.6	7.3	7.3 - 8.1	6.6	7.3	5.1 - 6.8	5.1 - 7.5	6.2 - 7.7	7.7	*

B.

**Group II species**	_C. abies-marina_	_Cystoseira_ sp. MP14*	_C. sonderi_	_C. squarrosa_*	_C. elegans_*	_C. baccata_	_C. barbata_	_C. mauritanica_*	_C. barbata_ f. _aurantia_*	_C. elegans_*	_C. granulata_*	_C. nodicaulis_	_Cystoseira_ sp. 2*	_C. montagnei_	_C. montagnei_ var. _tenuior_*	_C. usneoides_
C. abies-marina	0.0 - 0.4															
Cystoseira sp. MP14*	0.0 - 0.7	*														
C. sonderi	2.4 - 3.5	2.7 - 2.4	0.0													
_C. squarrosa_*	11.1 - 12.0	11.1	9.3	*												
_C. elegans_*	11.5 - 12.4	11.5	9.7	0.3	*											
C. baccata	11.7 – 13.1	11.2 - 12.1	9.8 - 10.4	7.3 - 8.1	6.9 - 7.6	0.0										
C. barbata	12.0 – 13.8	11.5 - 12.9	9.7 - 11.5	3.9 - 4.7	1.7 - 4.3	5.4 - 7.2	0.7 - 3.9									
_C. mauritanica_*	11.1 - 12.0	11.1	9.7	2.1	1.7	6.5 - 7.2	0.0 - 3.9	*								
C. barbata f. _aurantia_*	11.5 - 12.4	11.5	9.7	2.1	1.7	6.5 - 7.2	0.0 - 3.9	0.0	*							
_C. elegans_*	10.9 - 11.8	10.9	9.5	1.8	1.4	6.7 - 7.4	1.1 - 3.3	1.1	1.1	*						
_C. granulata_*	11.6 - 12.5	11.6	10.2	2.1	1.7	6.9 - 7.7	0.7 - 3.9	0.7	0.7	1.1	*					
C. nodicaulis	11.5 - 14.0	11.5 – 13.5	10.1 - 11.8	2.1 - 2.4	1.7 - 2.0	7.6 - 8.9	0.7 - 4.5	0.7 - 0.8	0.7 - 0.8	1.1 - 1.2	0.0	0.0				
Cystoseira sp. 2*	10.6 - 11.5	10.6	9.3	1.4	1.0	6.2 - 6.8	0.7 - 3.2	0.7	0.7	0.4	0.7	0.7 - 0.8	*			
C. montagnei	10.6 - 12.9	10.6 - 12.0	9.3 - 10.1	0.7-1.4	0.3 - 1.0	6.2 - 8.1	0.7 - 4.7	0.7 - 2.1	0.7 - 2.1	0.4 - 1.8	0.7 - 2.1	0.7 - 2.4	0.0 - 1.4	0.0 - 1.4		
_C. montagnei_var._tenuior_*	10.6 - 11.5	10.6	9.3	1.4	1.0	6.2 - 6.8	0.7 - 3.2	0.7	0.7	0.4	0.7	0.7 - 0.8	0.0	0.0-1.4	*	
C. usneoides	12.0 - 12.9	11.5 - 12.0	9.9 - 11.0	4.5 - 4.9	4.1 - 4.5	4.0 - 4.5	2.2 - 4.1	3.7 - 4.1	3.7 - 4.1	3.4 - 3.8	3.3 - 3.7	3.3 - 4.2	2.9 - 3.3	2.9 – 4.4	2.9 - 3.3	0.0 - 0.4

C.

**Group III species**	_C. compressa_	_C. compressa_ subsp. _pustulata_*	_C. humilis_ var. _myriophylloides_*	_Cystoseira sp._ MP2*	_Cystoseira_ sp. MP1*	_C. foeniculacea_
C. compressa	0.4 - 0.8					
C. compressa subsp. _pustulata_*	0.7 - 1.5	*				
C. humilis var. _myriophylloides_*	0.4 - 0.8	0.4 - 0.8	*			
Cystoseira sp. MP2*	1.5 - 1.9	1.5 - 1.9	1.1	*		
Cystoseira sp. MP1*	1.5 - 1.9	1.5 - 1.9	1.1	0.0	*	
C. foeniculacea	9.7 - 10.9	8.8 - 9.3	9.3 - 9.9	10.6 - 11.4	10.6 - 11.4	0.0

* Species represented by only one specimen
