# Supplementary material for: Improved phylogeny of brown algae Cystoseira (Fucales) from the Atlantic-Mediterranean region based on mitochondrial sequences
Source: PLoS One. 2019 Jan 30;14(1):e0210143. doi: 10.1371/journal.pone.0210143 (PMC6364706; doi:10.1371/journal.pone.0210143)
Supplement: S5 Table — (PDF) [file pone.0210143.s005.pdf]

S5 Table. Morphological traits identified by other authors for the different *Cystoseira* phylogenetic groups included in this study.

Taxa[1]	This study	Phylogenetic Group*	Embryo			Conceptacle structure		Antherozoid
			Oosphere shape	Segmentation sequence	Primary rhizoid number	Antheridia branches	Trichothallic growth	
C. amentacea, *C. mediterranea*, *C. tamariscifolia*	Cystoseira-IA	I / I	Spherical	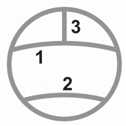	4	Numerous and branched	Absent	with stigma
C. brachycarpa, *C. crinita*	Cystoseira-IB	II / I						
C. zosteroides	Cystoseira-IC	III / I						
C. elegans, *C. nodicaulis*, *C. montagnei*, *C. mauritanica*, *C. squarrosa*, *C. usneoides*	Cystoseira-IIA	III / I-n.d.	Spherical	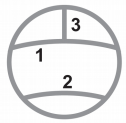	4	Numerous and branched	Absent	with stigma
C. barbata	Cystoseira-IIA	II / I–n.d						
C. abies-marina	Cystoseira-IIB							
C. baccata	Cystoseira-IIA	VI / II		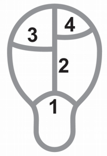				
C. compressa, *C. humilis*, *C. foeniculacea*	Cystoseira-IIIA	IV-V / III	Ovoid	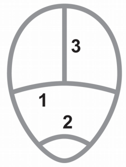	8	Few and branched	Present	without stigma

1Conspecifity of taxa used by different authors [40]: *C. amentacea* = *C. stricta*; *C. brachycarpa* = *C. balearica* = *C. caespitosa*; *C. barbata* = *C. susanensis*; *C. nodicaulis* = *C. granulata*; *C. montagnei* = *C. spinosa* = *C. jabukae*; *C. squarrosa* = *C. spinosa var. squarrosa*; *C. foeniculacea* = *C. Ergovicii*; n.d. – not determined; * Groups as defined by Amico et al. [27] / Colombo et al. [80]
