# Supplementary figures and images for: Improved phylogeny of brown algae Cystoseira (Fucales) from the Atlantic-Mediterranean region based on mitochondrial sequences
Source: PLoS One. 2019 Jan 30;14(1):e0210143. doi: 10.1371/journal.pone.0210143 (PMC6364706; doi:10.1371/journal.pone.0210143)

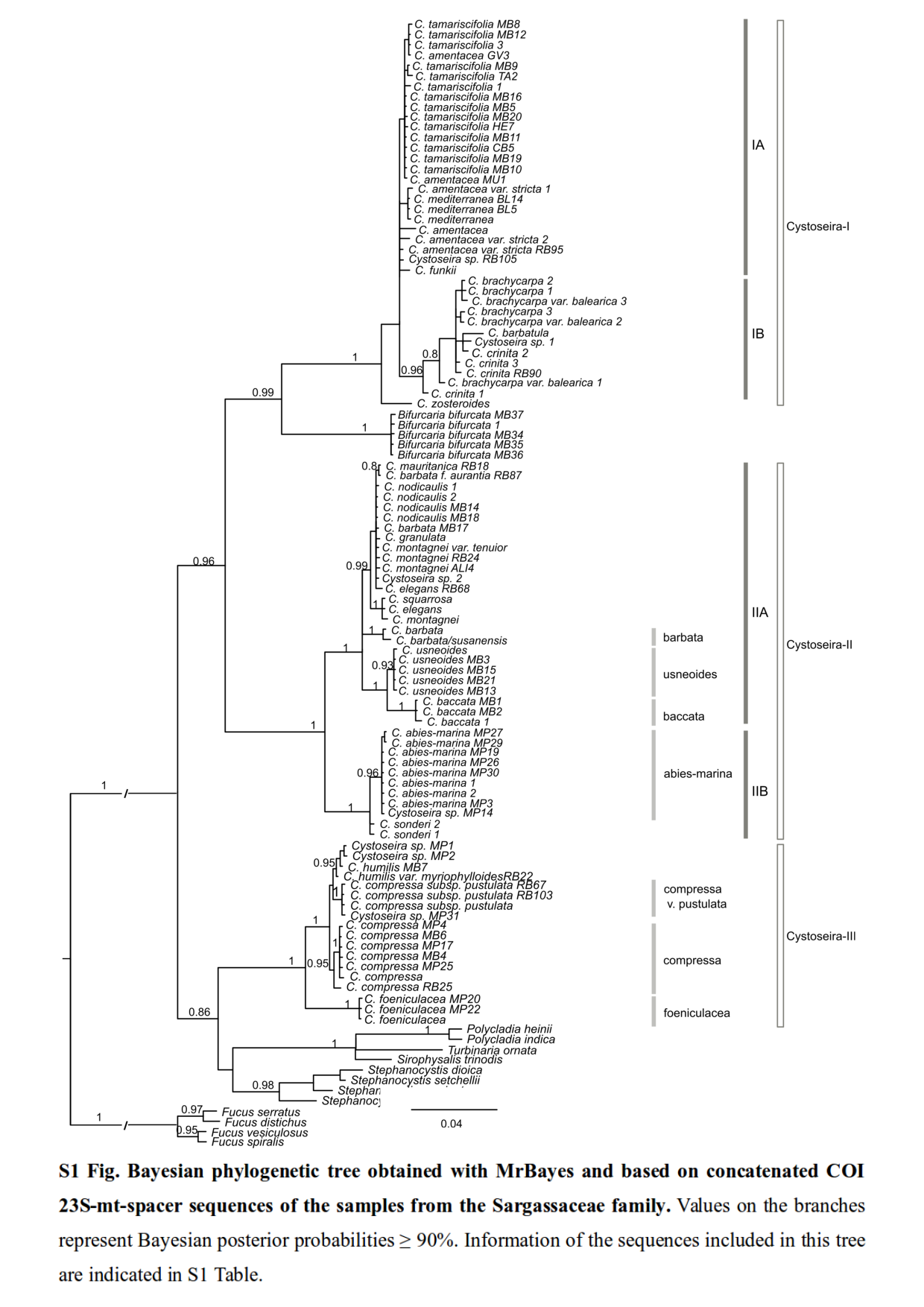

Supplement: S1 Fig — Values on the branches represent Bayesian posterior probabilities ≥ 90%. Information of the sequences included in this tree are indicated in S1 Table. (PNG) [file pone.0210143.s006.png]

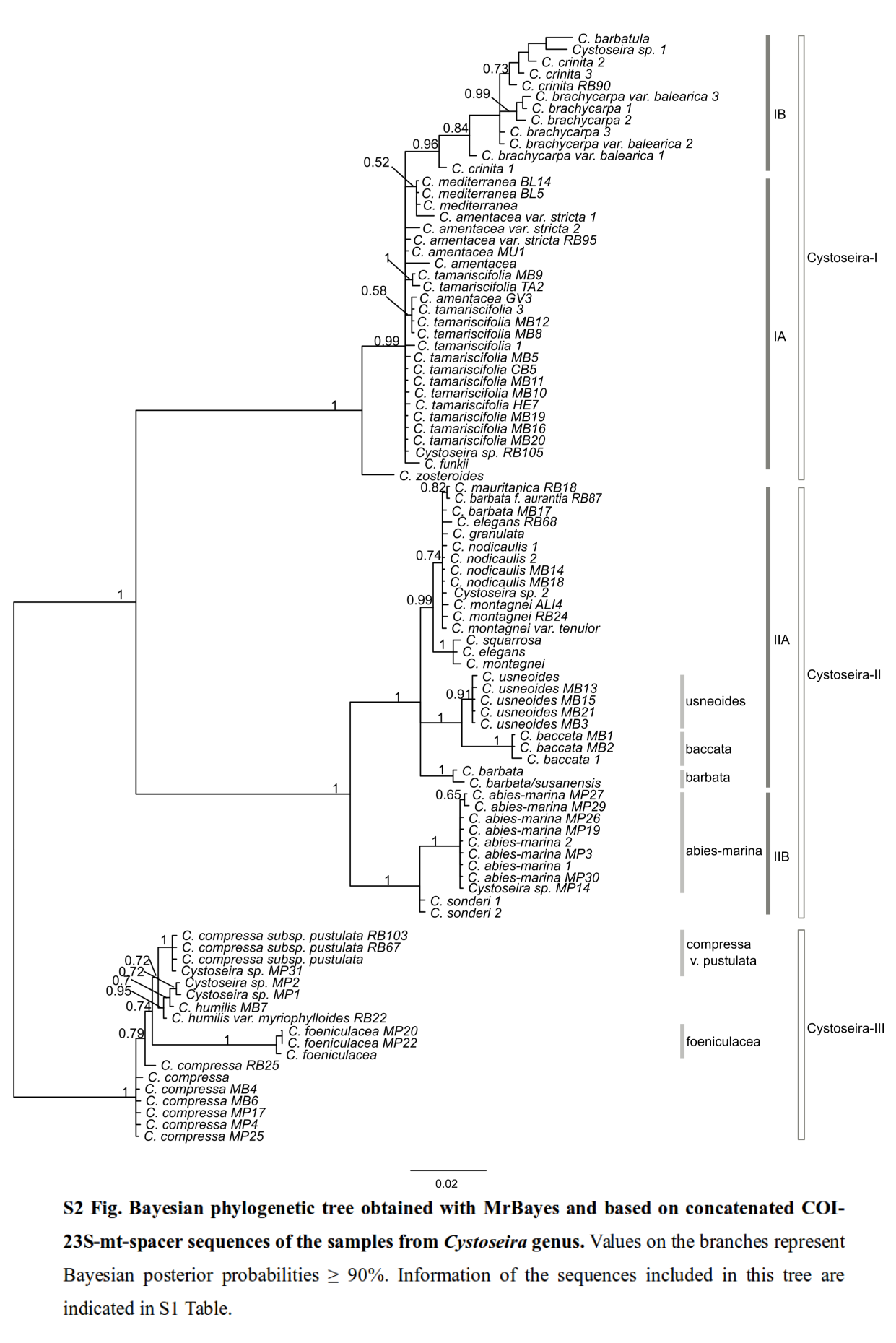

Supplement: S2 Fig — Values on the branches represent Bayesian posterior probabilities ≥ 90%. Information of the sequences included in this tree are indicated in S1 Table. (PNG) [file pone.0210143.s007.png]

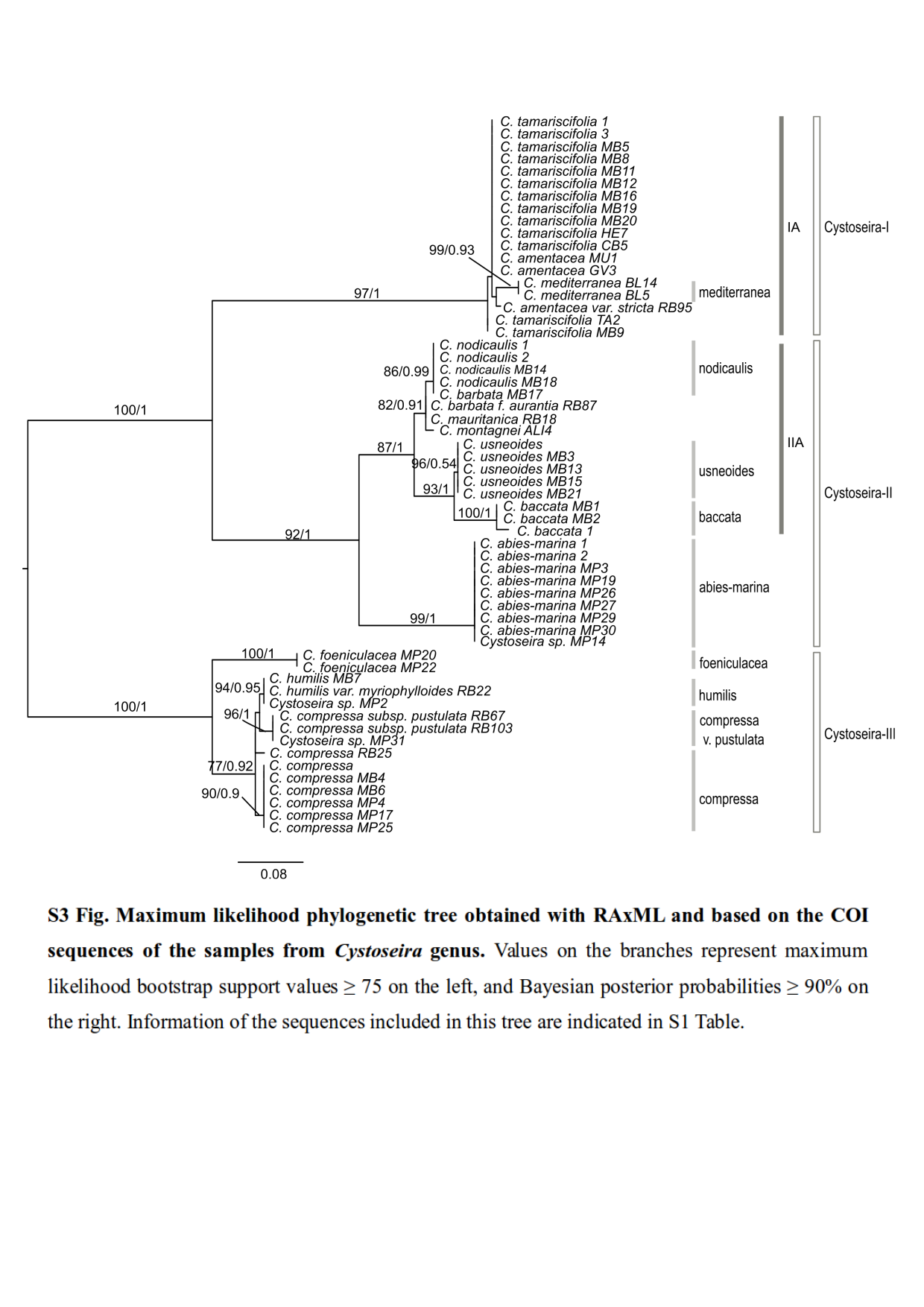

Supplement: S3 Fig — Values on the branches represent maximum likelihood bootstrap support values ≥ 75 on the left, and Bayesian posterior probabilities ≥ 90% on the right. Information of the sequences included in this tree are indicated in S1 Table. (PNG) [file pone.0210143.s008.png]

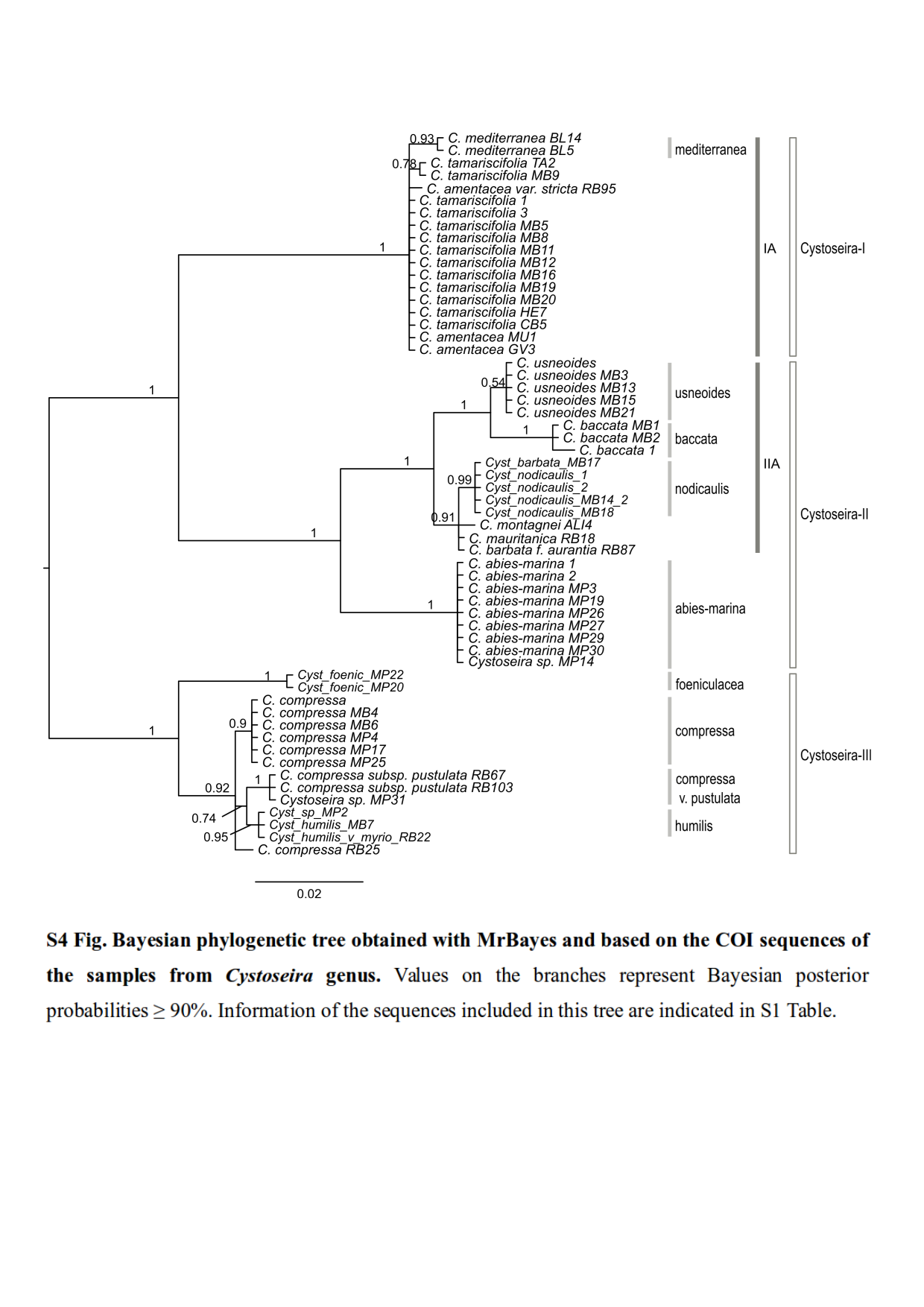

Supplement: S4 Fig — Values on the branches represent Bayesian posterior probabilities ≥ 90%. Information of the sequences included in this tree are indicated in S1 Table. (PNG) [file pone.0210143.s009.png]

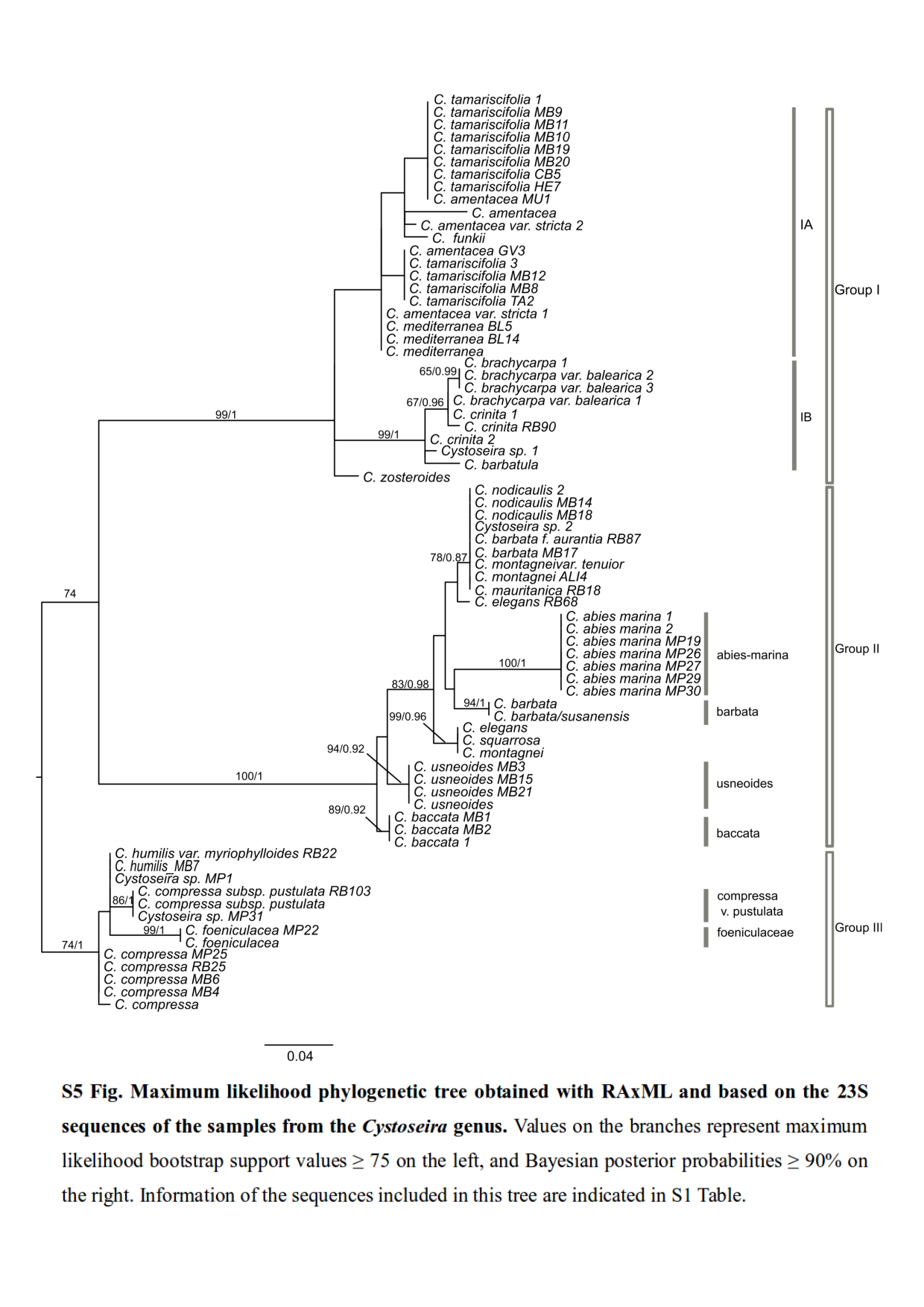

Supplement: S5 Fig — Values on the branches represent maximum likelihood bootstrap support values ≥ 75 on the left, and Bayesian posterior probabilities ≥ 90% on the right. Information of the sequences included in this tree are indicated in S1 Table. (PNG) [file pone.0210143.s010.png]

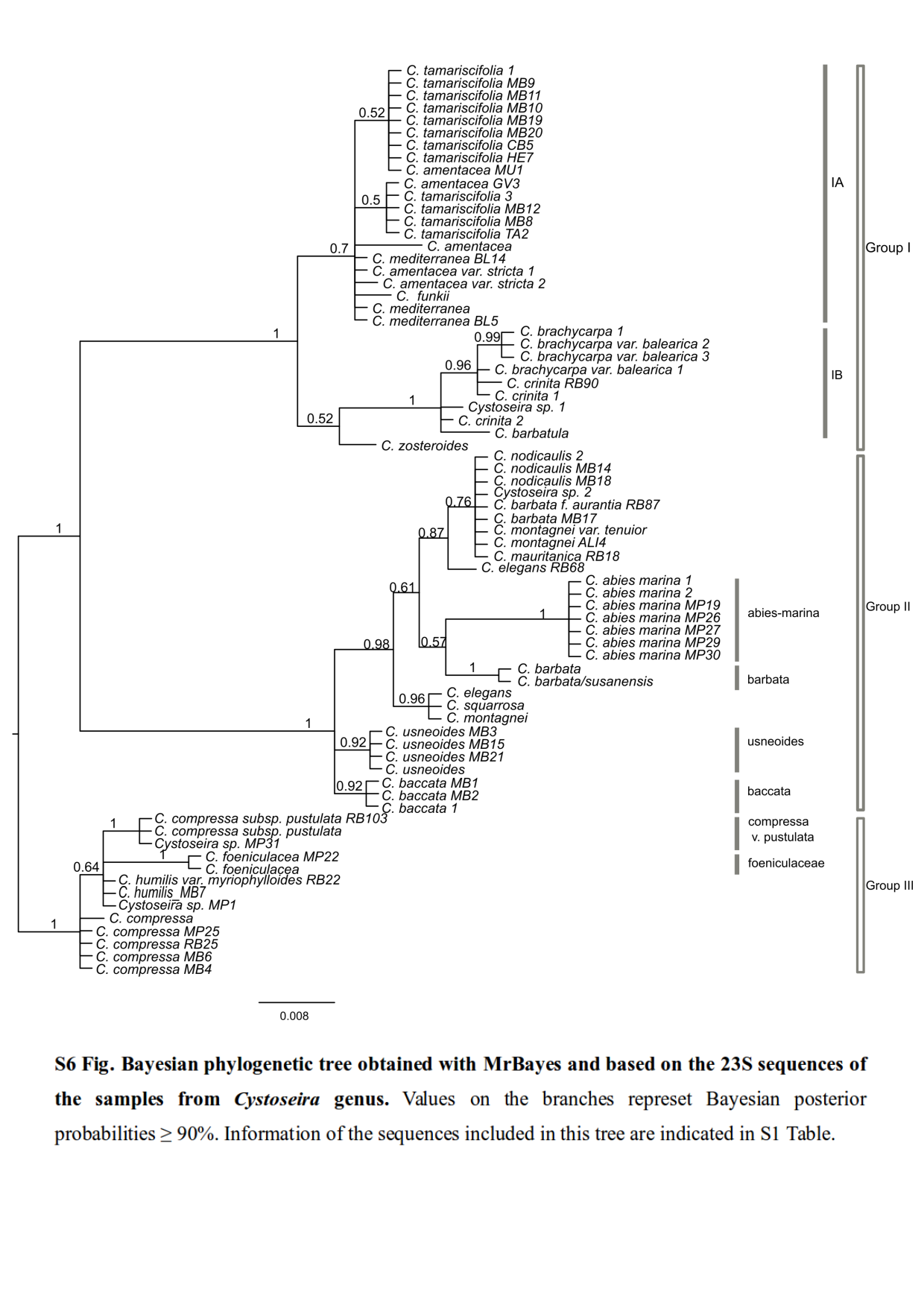

Supplement: S6 Fig — Values on the branches represet Bayesian posterior probabilities ≥ 90%. Information of the sequences included in this tree are indicated in S1 Table. (PNG) [file pone.0210143.s011.png]

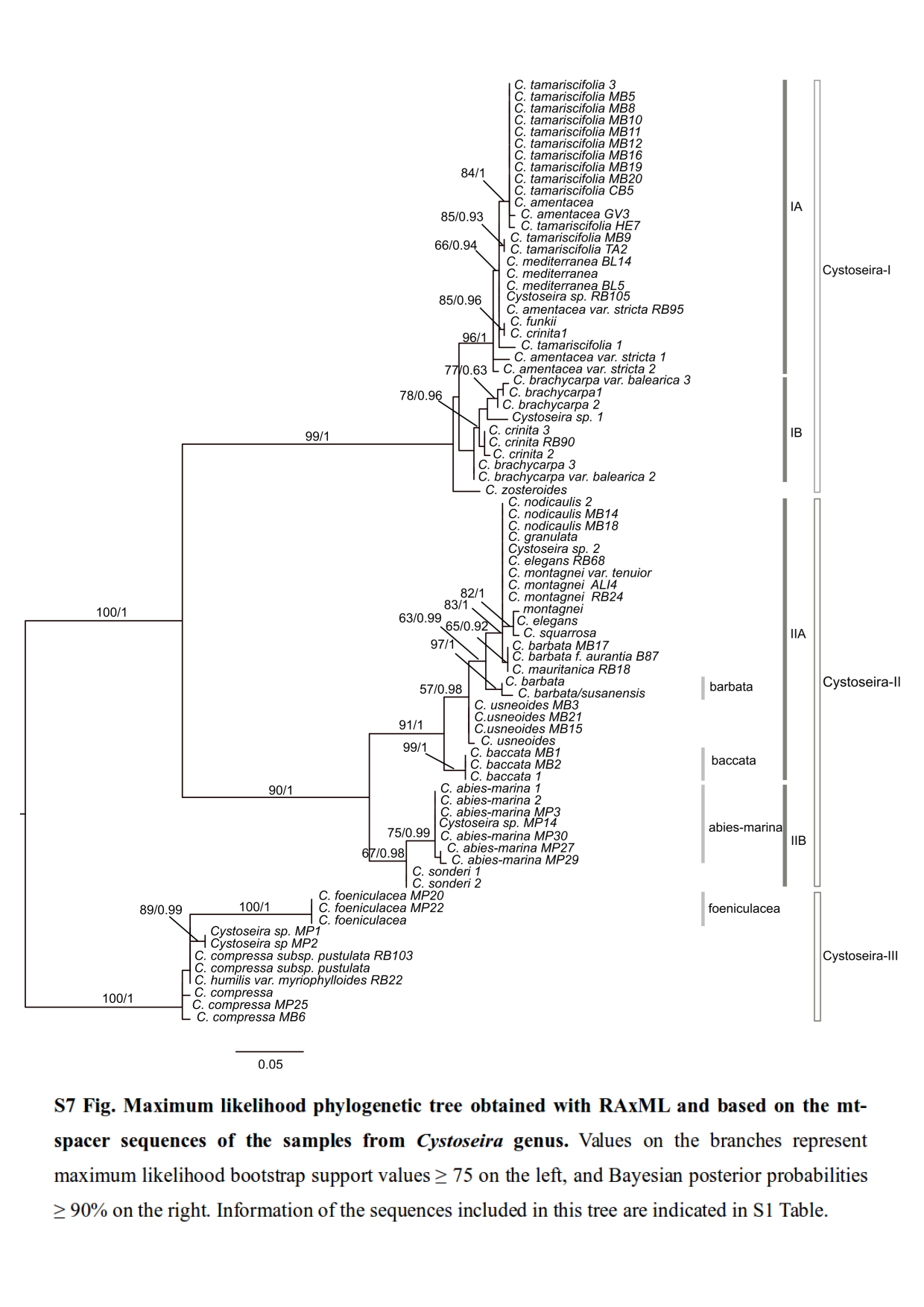

Supplement: S7 Fig — Values on the branches represent maximum likelihood bootstrap support values ≥ 75 on the left, and Bayesian posterior probabilities ≥ 90% on the right. Information of the sequences included in this tree are indicated in S1 Table. (PNG) [file pone.0210143.s012.png]

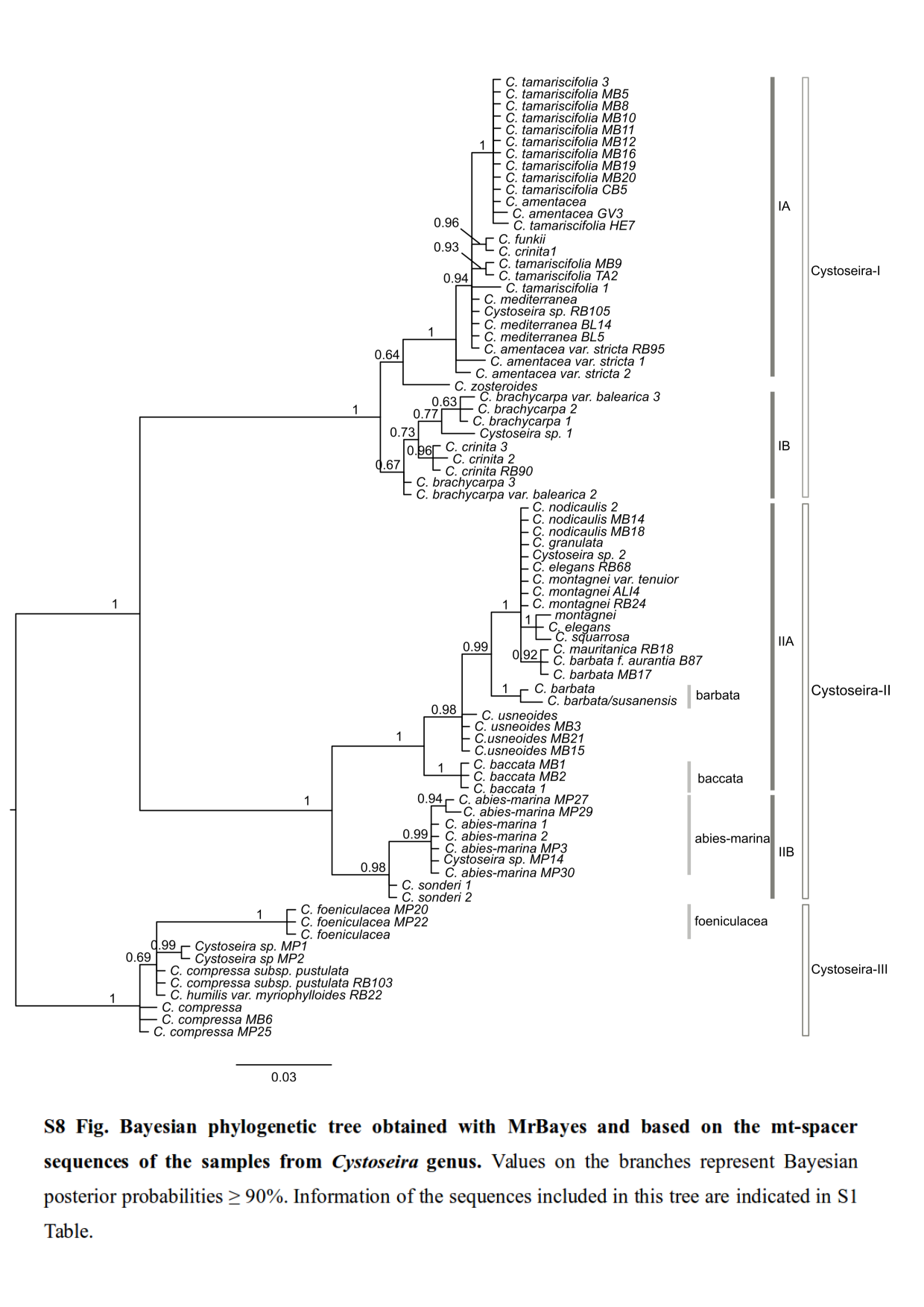

Supplement: S8 Fig — Values on the branches represent Bayesian posterior probabilities ≥ 90%. Information of the sequences included in this tree are indicated in S1 Table. (PNG) [file pone.0210143.s013.png]

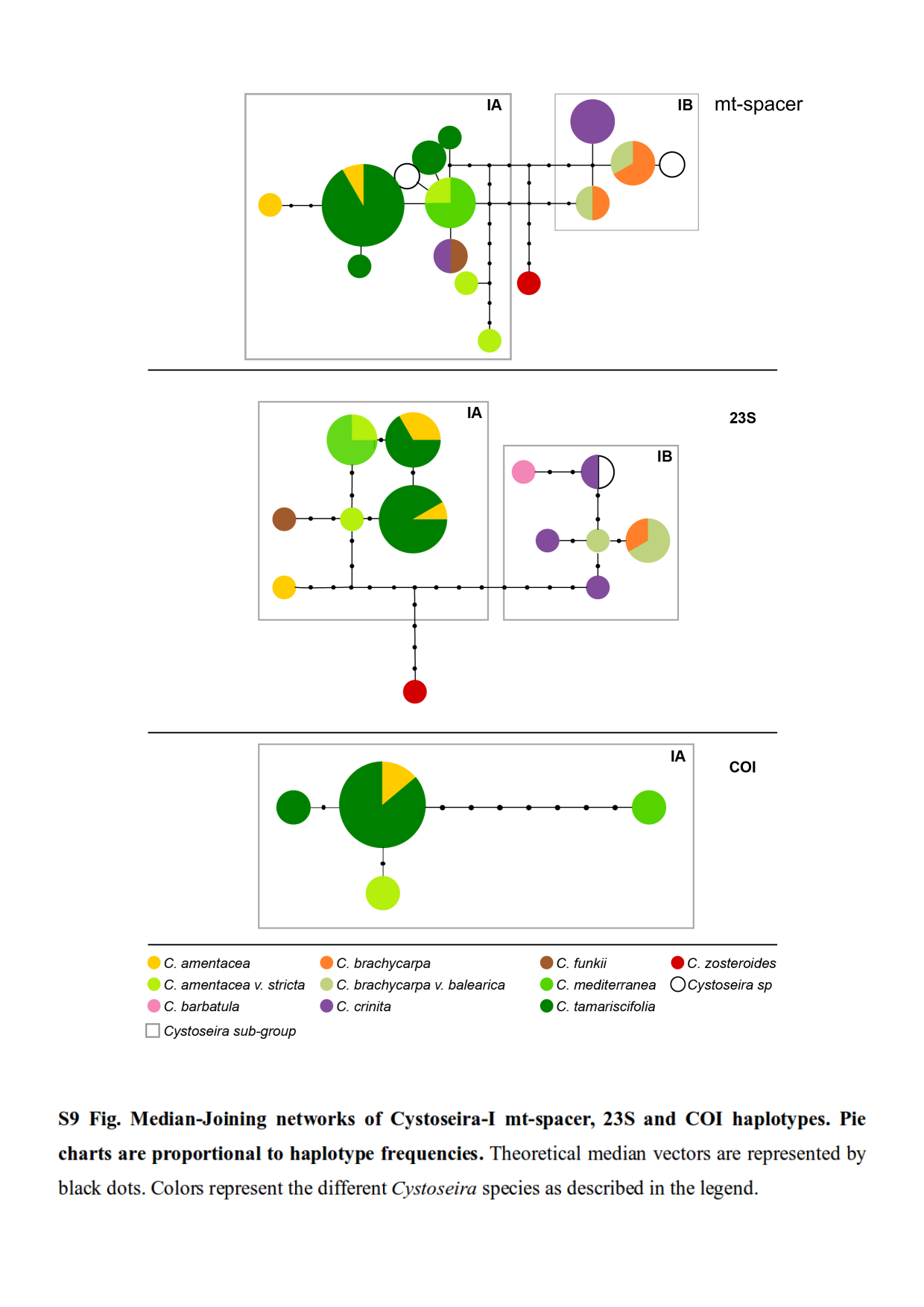

Supplement: S9 Fig — Pie charts are proportional to haplotype frequencies. Theoretical median vectors are represented by black dots. Colors represent the different Cystoseira species as described in the legend. (PNG) [file pone.0210143.s014.png]

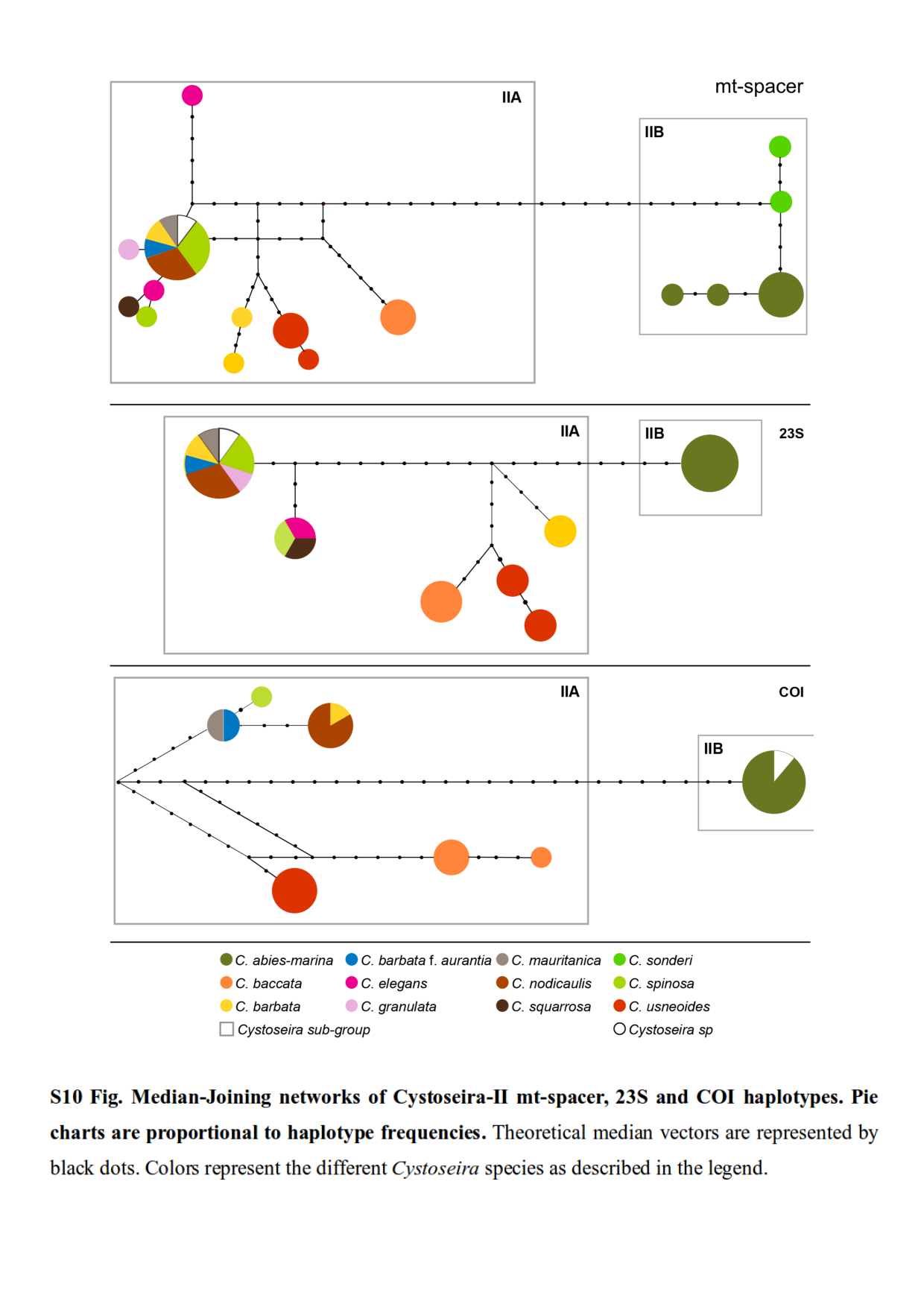

Supplement: S10 Fig — Pie charts are proportional to haplotype frequencies. Theoretical median vectors are represented by black dots. Colors represent the different Cystoseira species as described in the legend. (PNG) [file pone.0210143.s015.png]

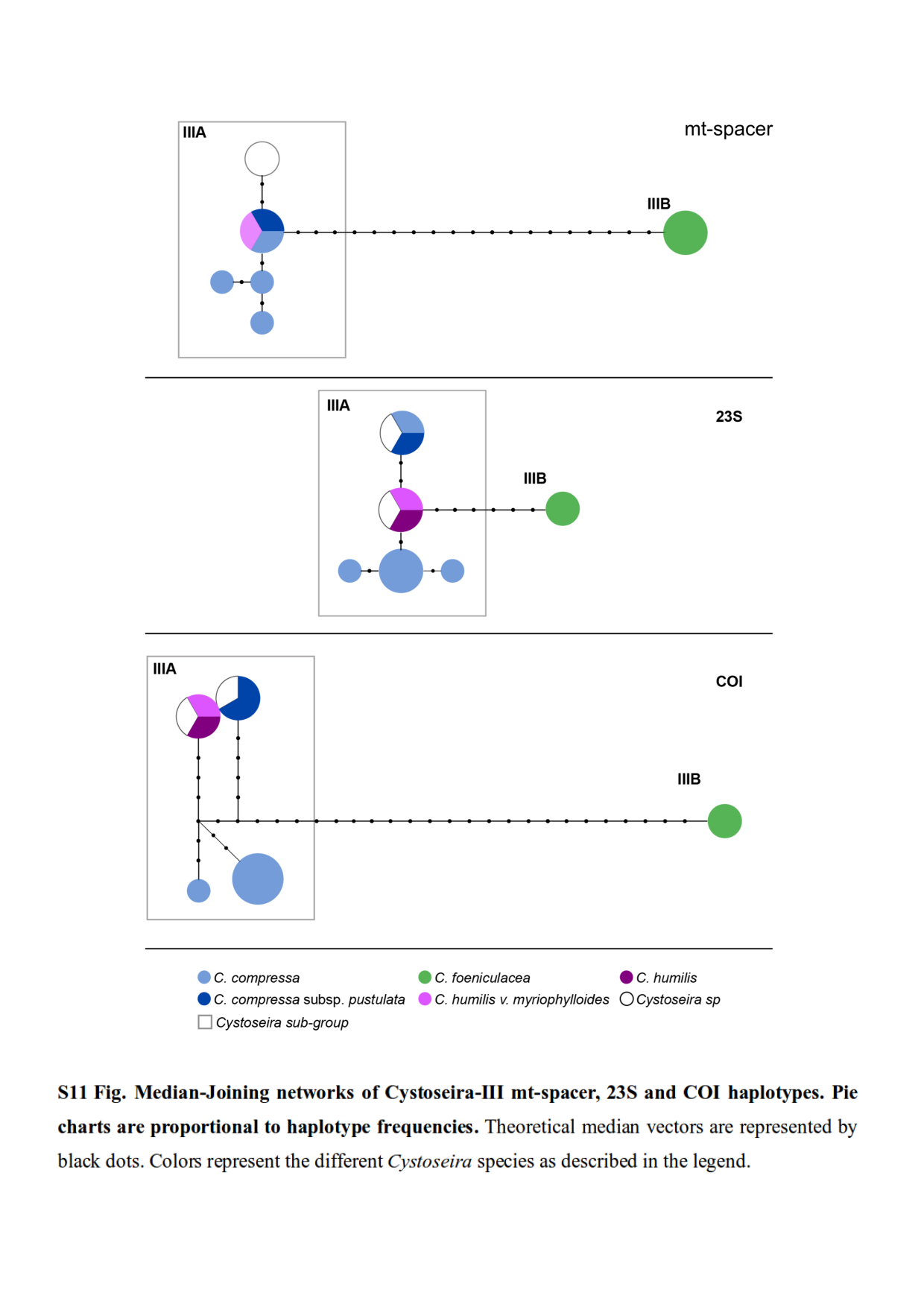

Supplement: S11 Fig — Pie charts are proportional to haplotype frequencies. Theoretical median vectors are represented by black dots. Colors represent the different Cystoseira species as described in the legend. (PNG) [file pone.0210143.s016.png]
